# Evolution of social relationships between first-year students at middle school: from cliques to circles

**DOI:** 10.1038/s41598-021-90984-z

**Published:** 2021-06-03

**Authors:** Diego Escribano, Victoria Doldán-Martelli, Francisco J. Lapuente, José A. Cuesta, Angel Sánchez

**Affiliations:** 1 Grupo Interdisciplinar de Sistemas Complejos, Departamento de Matemáticas, Universidad Carlos III de Madrid, 28911 Leganés, Madrid, Spain; 2 Unidad Mixta Interdisciplinar de Comportamiento y Unidad Social (UMICSS) UC3M-UV-UZ, Universidad Carlos III de Madrid, 28911 Leganés, Spain; 3 Instituto de Enseñanza Secundaria Blas de Otero, 28024 Madrid, Spain; 4 Departamento de Biología y Geología, Física y Química Inorgánica, Universidad Rey Juan Carlos, 28933 Móstoles, Madrid, Spain; 5 Institute for Biocomputation and Physics of Complex Systems (BIFI), University of Zaragoza, 50018 Zaragoza, Spain; 6 UC3M-Santander Big Data Institute (IBiDat), 28903 Getafe, Spain

**Keywords:** Applied mathematics, Complex networks

## Abstract

People organize their social relationships under a restriction on the number that a single individual can maintain simultaneously (the so-called Dunbar’s number, ~150). Additionally, personal networks show a characteristic layered structure where each layer corresponds to relationships of different emotional closeness. This structure, referred to as Dunbar’s circles, has mostly been considered from a static viewpoint, and their structure and evolution is largely unexplored. Here we study the issue of the evolution of the structure of positive and negative relationships in early adolescence by using data from students in their first year at middle school obtained from surveys conducted in class in two different waves separated by several months. Our results show that, initially, students have a lower number of total relationships but the majority are more intense and over time they report a higher number of total relationships, but the more intense relationships appear in a lower proportion. We have also found differences in the structure of communities at both temporal moments. While in the first instance the communities that appeared are mixed, made up of both boys and girls, in the second they changed so that they were separated primarily by gender. In addition, the size of each community was stabilized around 15 people, which coincides with the size of the second Dunbar’s circle, known as the sympathy group in social psychology. As a consequence, in groups with around 20 students of the same gender, they tend to split in two separate communities of about 10 each, below the second Dunbar’s circle threshold. On the other hand, groups with more stable community structure appear to go through the inverse process of friendship evolution, becoming more focused on their best relationships. All these results suggest how the layered structure of the personal network, as well as the community structure of the social network, emerge directly from the union of both positive and negative relationships. Thus, we provide a new perspective about its temporal evolution that may have relevant applications to improve school life and student performance.

## Introduction

Over the past twenty years^1^, research has accumulated evidence showing that the number and quality of our social relationships play a key role in our happiness^2^, our mental and physical health^3, 4^ and our social capital^5, 6^. The organization of our friendships and acquaintances is also crucial to understand how contagious diseases propagate and can be contained^7, 8^. Similar effects arise also when considering information or opinion diffusion^9^ and social norm formation^10^. Social network analysis, a discipline combining the insights of sociology and complex systems and networks, is therefore instrumental to advance our knowledge of these phenomena^11, 12^. In this regard, a key finding is that the average size of personal social networks is approximately 150^13–15^, with typical sizes varying in the range 78–250^16^. These networks actually consist of a series of layers that correspond to relationships of different quality^13, 17, 18^. The cumulative account of individuals up to a certain layer is what is referred to in the literature as ‘circles’. Circles exhibit distinct sizes (around 1.5, 5, 15, 50, and 150), i.e., each circle is roughly three times the size of the previous one. The different layers could be characterized as primary partner(s), intimate, best and good friends and, finally, just friends, constituting the so called ‘active network’^16^. Sociological research on strong and weak ties suggests that the 5-circle is in fact a support network^18^, while the 15-circle is known in social psychology as a sympathy group, consisting of people whose death would cause great distress^19^. Recently, it has been shown^20^ that the existence of circles and of a scaling ratio between them is a consequence of the limitations of our cognitive capacity and of the different time devoted to friendships of different emotional closeness.

While the above framework has proven itself both accurate and useful, there are two main issues that require further research to better benefit from its application to social phenomena. First, the static picture of the layers must be scaled to take into account temporal effects and its dynamical evolution^21^. There are some studies incorporating the time dimension to the data collection process, either for short time-scales^22, 23^ or for longer ones^24, 25^. However, much more research is needed to characterize in detail the evolution of friendships and, in particular, to shed light on the relevant mechanisms driving it. Second, the size and structure of personal networks is subject to strong age effects^13, 26, 27^. 20-year-old people usually have more friends than 60-year-old ones, mostly because the elderly tend to have outside layers with lower numbers of friends^28^. Middle age is characterized as a stability period until a rapid declining in the number of friendships sets in^27, 29^. In turn, adolescents have very sensitive networks, suffering dramatic modifications when changing homes^30^ or starting college^31^.

In this paper, we study the issue of the evolution of friendships in early adolescence by using data from high school students. In contrast to previous research^23, 32–35^, here we focus on the existence and evolution of a Dunbar's circle structure in the students’ personal networks. To that end, we carried out two survey waves in different moments of the academic year, so we could compare the resulting networks. In addition, we include in our study not only positive relationships (friendships) but also negative ones (enmities). Due to the scarcity of negative links, the existence of Dunbar-like circles could be assessed for positive relationships only. However, a proper study of group or community-formation process in classes requires taking into account negative links as well. Indeed, community detection algorithms based on positive links only typically split a network into subgraphs or modules characterized by high link density^36^. However, in a social group such as a school class, where students interact daily for many hours, it seems only natural to consider the possibility that some students have bad relationships with others. It is clear that this could distort the picture arising from looking only at the positive relationships and, as a consequence, new community detection algorithms must be applied^37^.

## Results

### Individuals and Dunbar’s circles

We studied a group of students from a high school in Madrid, IES Blas de Otero, in the first year of secondary school (students between the ages of 12 and 13). Practically all students are new to the high school, having finished primary school somewhere else in the preceding year. A few students were taking first year again having failed the year before. Two data collection sessions were held respectively in December 2018 (wave 1) and May 2019 (wave 2). 151 students (73 boys and 78 girls, cf. Supplementary Information for more disaggregated numbers) and their families agreed to participate. The percentage of participation was 97% in the first wave and 90% in the second wave.

Students were born in 2006 (75%), 2005 (23%) and 2004 (3%). Classes were organized in 5 groups and were all enrolled at first year of ESO (mandatory secondary education, similar to middle school). Groups were labeled with letters, from A to E. Groups A and B received most of their teaching in English (all subjects except Math, Spanish and the chosen option between Religion and Ethical Values), whereas groups C through E were taught in Spanish except in Arts and Physical Education, where classes took place in English.

**Table 1:** Questionnaire for positive and negative relationships. Students could choose names among all other students in their year, by clicking on each of the groups.

**Questions regarding positive relationships**	
Question 1: Who are your friends inside the school?	
Question 2: Considering your friends: Who do you have the closest relationship with?	
Question 3: Finally, among your closest friends: who would you say are your best friends?	
(We are referring to those people with whom you are “flesh and bone”).	
**Questions regarding negative relationships**	
Question 4: Which partners do you not like at all or do not have a good relationship with?	
Question 5 : Considering the people you don’t like at all: who do you dislike or have problems with?	
Question 6 : Finally, considering the people you dislike: are there any people with whom you have a particularly bad or troubled relationship?	

Students were presented with a list of all other 150 participants and were asked a set of questions allowing us to infer positive and negative relationships with three levels of intensity (cf. Table 1). For various reasons, participants were not able to correctly discriminate between the two lower intensity levels, which were merged into a single level. Therefore, both positive and negative relationships were classified into two levels of intensity, which we will henceforth refer to as ‘friend’ and ‘best friend’ for positive relationships, and ‘enemy’ and ‘worst enemy’ for negative relationships. Data on the original answers prior to merging categories can be found in the Supplementary Information.

**Figure 1:**
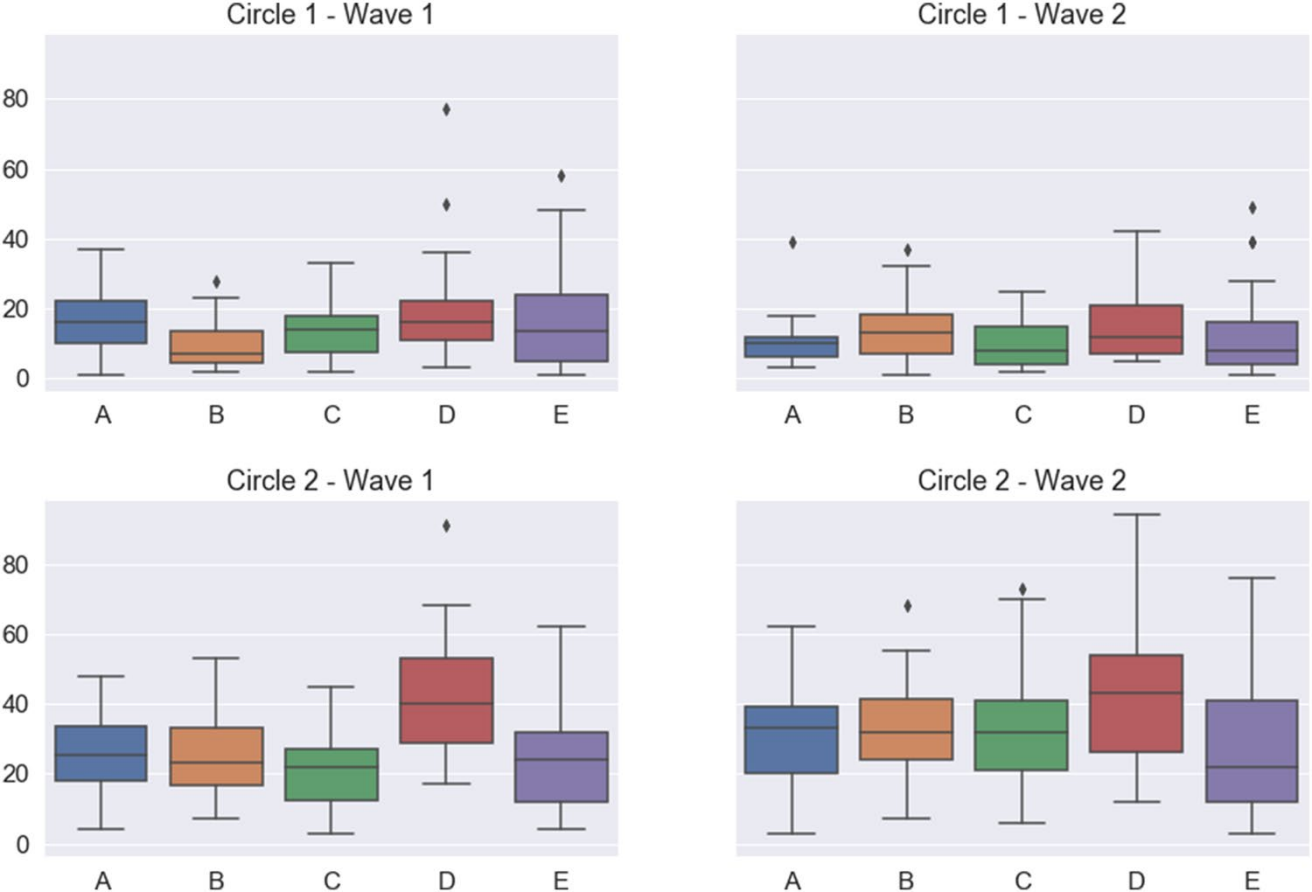
Boxplot of the answers about friendships and very good friendships. Top, numbers of students marked as “best friends” by students of each group (circle 1). Bottom, numbers of students marked as “friends” by students of each group (circle 2). Left, results from wave 1. Right, results from wave 2. Line represents the median, boxes the first and third quartiles, lines, the 95% confidence interval, and dots, outliers.

Our first result concerns the composition of the two circles of positive relationships and the comparison to the expected Dunbar’s circle structure. As can be seen from Fig. 1, the basic structure of the first two circles appears, with average sizes that vary considerably across groups. In wave 1, the global average over all participants in the survey yields a mean size for the “best friends” circle (hereafter, circle 1) of 14.48 and for the “friends” circle (hereafter, circle 2) of 27.01 which are clearly larger than the typical sizes reported for these two circles. In wave 2, these values become 11.27 and 28.47, respectively, still far from the expected values. There are several possible explanations for this finding. A first one is that students are still in an exploring phase, they have already established several relationships with people they did not know from school, while keeping friends with other students coming from their same school. Unfortunately, the onset of the Covid-19 pandemics prevented us from continuing our historical series of waves when schools were put under lockdown, so we cannot say much more about the evolution of friendships. Another possibility is that our questions are in fact not eliciting circles 1 and 2, but instead circles 2 and 3. In that case, the results would be more in line with the expected Dunbar's circle structure (albeit circle 3 might be not entirely elicited), but we cannot be sure whether students interpreted our questions in that manner.

**Figure 2:**
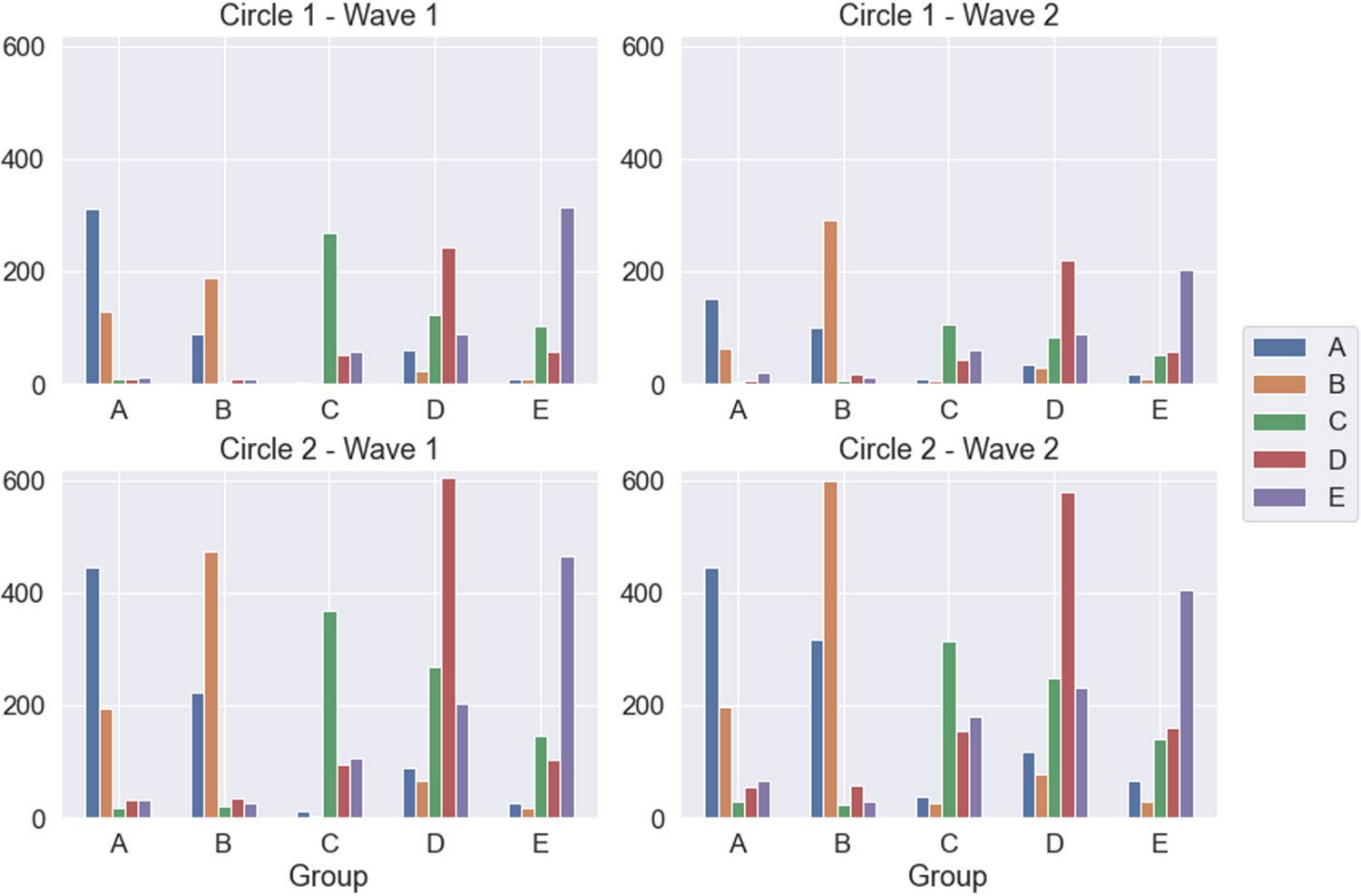
Distribution of relationships among groups. Left: total number of students marked in wave 1, separated by group of the student named. Right: same for wave 2. Top: circle 1. Bottom: circle 2. Colors indicate the group where the students with whom the relationships are reported belong (color code on the right of the figure).

**Figure 3:**
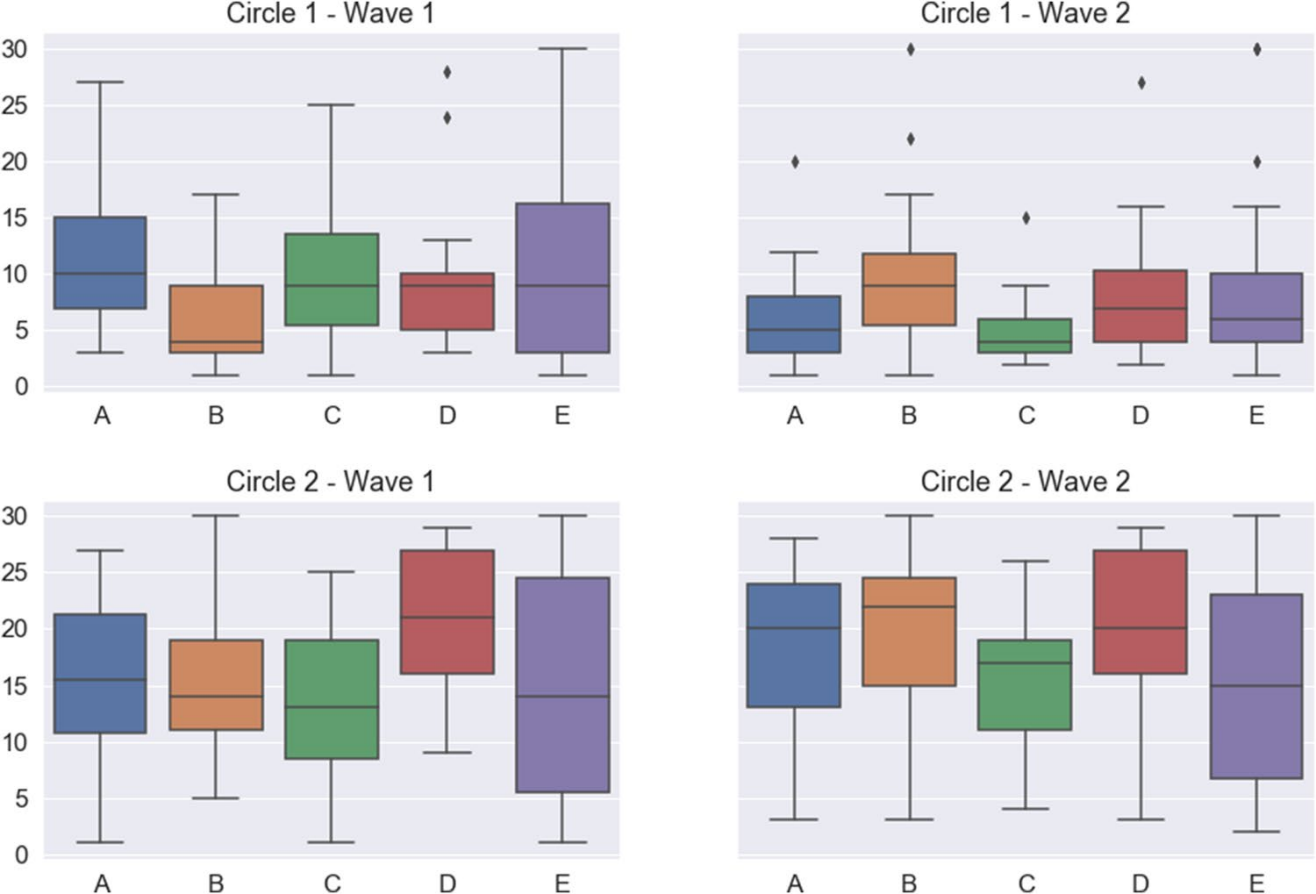
Boxplot of the answers about friendships and very good friendships restricted to the ego’s group. Top, numbers of students marked as “best friends” by students of each group (circle 1). Bottom, numbers of students marked as “friends” by students of each group (circle 2). Only relationships within the group are shown. Left, results from wave 1. Right, results from wave 2. Line represents the median, boxes the first and third quartiles, lines, the 95% confidence interval, and dots, outliers.

Another hypothesis that may explain the large observed values is that students treat relationships in their group differently from those with other groups, thus devoting separate cognitive efforts to each set. For each group, the majority of the relationships reported (67.22% in wave 1, 56.98% in wave 2) are with students in the same group (cf. Fig. 2). It is interesting to note that students in the English groups (A and B) declare practically no relationship with students in the Spanish groups (C, D, E) in wave 1, and only a few more in wave 2, and vice-versa. This may have to do with their primary schools of origin, as English groups can only be joined by students coming from English speaking primary schools and practically all of them come from just two such schools. If we restrict ourselves to intra-group relationships, the average sizes of the circles become 8.79 and 15.64 in wave 1 and 6.47 and 15.56 (cf. Fig. 3), much closer to the typical values for the first Dunbar's circles, which would suggest that the hypothesis advanced at the beginning of the paragraph may have some truth to it.

As for reported negative relationships, their absolute number is much smaller than that of positive ones, with average values of 4.82 for bad relationships and 2.07 for very bad ones (7.83 and 2.11 in wave 2; plots for negative relationships are included in the Supplementary Information). Even if there are not that many negative links, it is important to note that while the number of very bad relationships remains constant, there is a relatively large increase of the number of bad relationships from wave 1 to wave 2.

**Figure 4:**
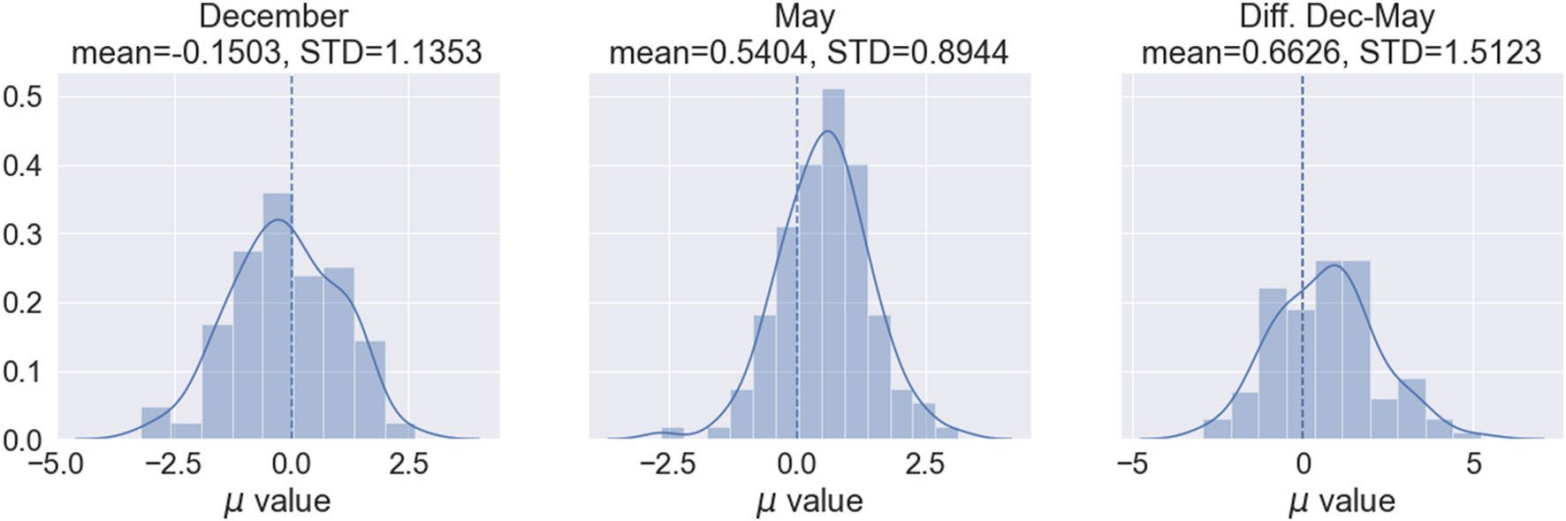
Distribution of $\mu$ values for all the participants in (**a**) December 2018 (wave 1), (**b**) May 2019 (wave 2) and (**c**) increment in the value of $\mu$ between wave 1 and wave 2. Data distribution fitted using a Kernel Density Estimation (KDE). Vertical dotted line shows $\mu$=0.

Looking now at the time evolution between the two waves, Figs. 1 and 2 along with the data above suggest a general trend in which the size of circle 1 decreases and the size of circle 2 remains constant or even increases. To analyze this possibility in more detail, we resorted to the use of the parameter $\mu$, which was introduced in Ref. ^20^ as a summary of the circle structure of a given individual (cf. Methods below for more details). In a nutshell, $\mu =\log \big ((C_2-C_1)/C_1\big )$, $C_i$ being the number of relationships in circle *i* (see Methods). When $\mu >0$ we have a typical structure of the circles, with the number of friendships in each one increasing rapidly as we move outside; a value $\mu \sim 0.7$ arises when the scaling ratio between circle sizes is around 3, as frequently observed. On the contrary, when $\mu <0$ most friendships are already in circle 1, with additional circles bringing in very few additional people. We fitted the analytical expression from Ref. ^20^ to the reported numbers of friendships in the two circles for each student, thus assigning them a specific value of $\mu$. Figure 4 shows the distribution of the values of $\mu$ in the two waves, and the distribution of their difference. It turns out that the average $\mu$ in wave 1 is positive only for groups B and D (cf. Table 2), while the increment in the value of $\mu$ between waves is positive in all cases except in group B. Further details about the changes of $\mu$ can be found in the plots in the Supplementary Information.

**Table 2:** Mean and standard deviation (SD) values for the $\mu$ parameter in December, May, and the increment in the $\mu$ value between December 2018 and May 2019.

Group	Mean(SD) wave 1	Mean (SD) wave 2	Mean(SD) increment
A	$-0.633$ (0.932)	0.782 (0.807)	1.399 (1.248)
B	0.499 (0.940)	0.362 (0.951)	$-0.137$ (1.222)
C	$-0.713$ (1.013)	0.788 (1.127)	1.515 (1.949)
D	0.352 (0.939)	0.560 (0.824)	0.208 (0.967)
E	$-0.573$ (1.232)	0.366 (0.750)	0.912 (1.588)
TOTAL	$-0.150$ (1.135)	0.540 (0.894)	0.663 (1.512)

**Figure 5:**
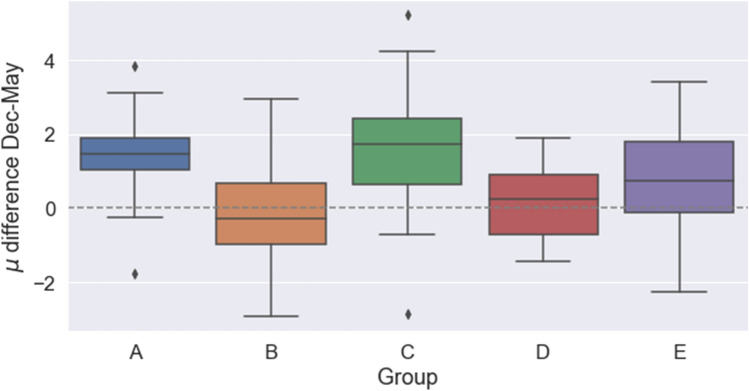
Boxplot of the increment in the $\mu$ parameter between December 2018 (wave 1) and May 2019 (wave 2), for each group. The difference of $\mu$ is normally distributed for all groups (Shapiro-Wilk test). Groups A, C and E show an increase in $\mu$, whereas for the other two groups the increment is not statistically different from zero (one-sample t-test).

Figure 5 shows the distribution of individual differences in the value of $\mu$ between waves, which is normally distributed for all groups (Shapiro-Wilk test). A one-sample t-test showed that the mean for the $\mu$ difference was statistically different from 0 only for groups A, C and E. A one-way ANOVA test determined that there was a statistically significant difference between groups ($F(4,117)=6.674$, $p<10^{-4}$). The Tukey HSD post hoc test showed that group B was significantly different to groups A, C and E, while group D was significantly different from groups A and C. Altogether, we can conclude that groups B, D and E, behave differently to groups A and C. The high frequency of cases in which $\mu$ decreases could indicate that these groups (B, D and E) have some particular feature which, if it exists, is not related to the teaching language, as both English- and Spanish-speaking show this behavior.

To gain further insight on this result, we resorted to consider the network of nominations received. Thus far, we have looked at friendships from the perspective of ego, i.e., the nominating student, considering as friends or best-friends all those they mentioned in the survey (outgoing links). The question we ask now is whether looking at the network from the perspective of being nominated, i.e, of the incoming links, we observe something similar to what we have noticed above. In this regard, it is interesting to note that in our results, 56.6% of the positive relationships are reciprocated in wave 1, and 58.3% in wave 2 (in line with the fact that surveys produce only approximately a 50% of reciprocated nominations^39, 40^). However, if we rewire the links in the network at random, the reciprocity is much smaller ($22.6\%$, CI: $(21.4\%; 23.8\%)$ in the network of wave 1, $24.6\%$, CI $(23.4\%; 25.8\%)$ in wave 2; see Supplementary Information for details of the comparison with the reciprocity of randomly rewirings of our networks), meaning that the observed reciprocity is large and that the network of incoming nominations may exhibit properties similar to the outgoing ones. To carry out the analysis, we introduce the parameter $\rho$, which plays the same role as $\mu$ but using in-degrees instead of out-degrees. The results of this analysis confirm the picture we have presented above, perhaps even more clearly (cf. figures in the Supplementary Information). We can thus say that most students in groups A and C, and a majority of those in the rest of the groups, change from having mostly best friends and only a few friends to a more standard circle structure with less best friends and more friends. Finally, we have not carried out a similar study with negative relationships because the small number of them prevents us from extracting meaningful values for $\mu$ or $\rho$ for enmities.

### Networks and community structure

**Figure 6:**
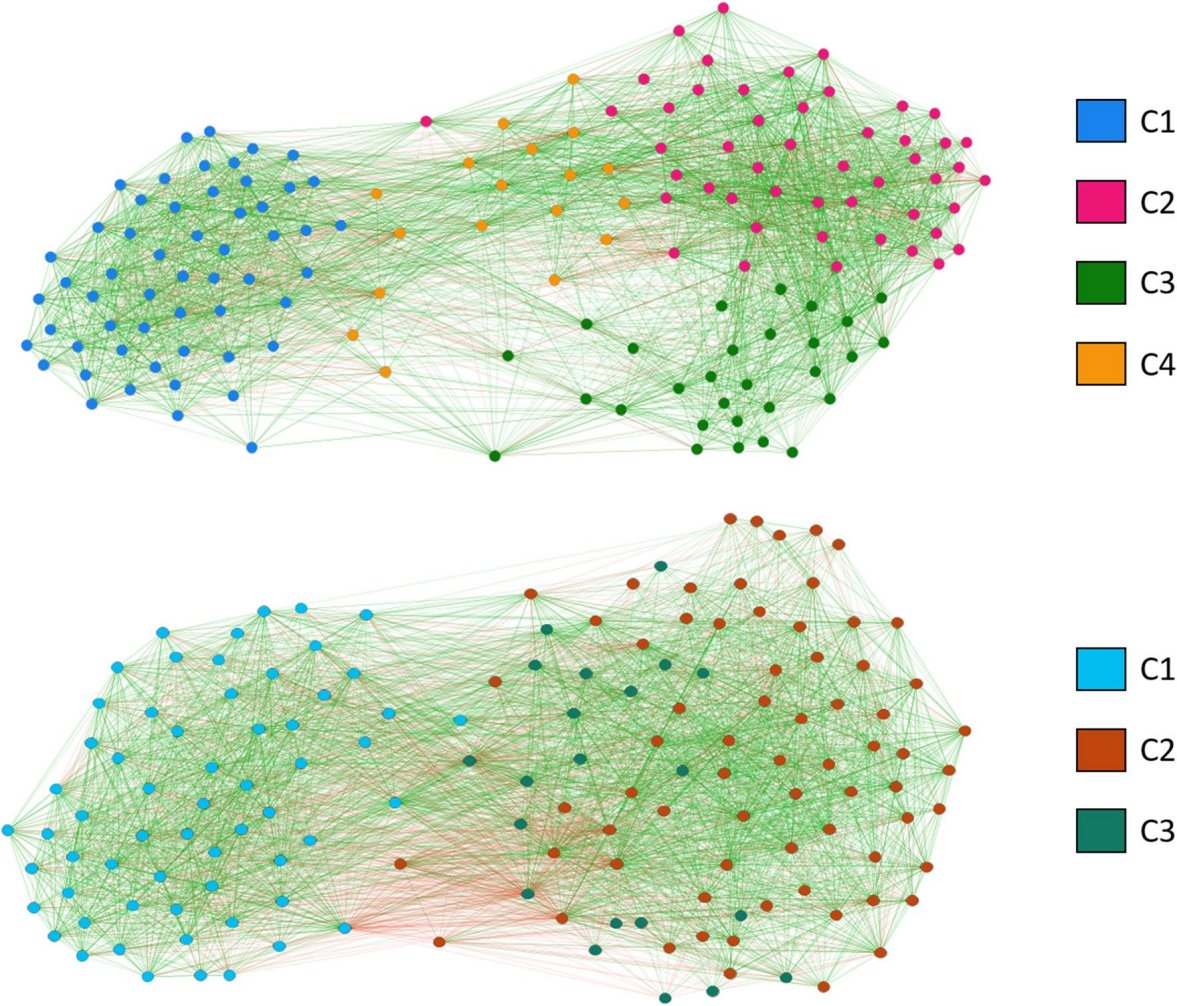
Network representation of all the social relations in wave 1 (top) and wave 2 (bottom). Nodes belonging to each community are marked by color as indicated in the plot. Friendship links are drawn in green, whereas enmity links are drawn in red.

Beyond the evolution they reflect at the level of individual students, changes in $\mu$ might be also reflecting structural changes in the social network. In order to assess this possibility, we have carried out a study of the structure of the groups by means of community analysis (see Methods below for a discussion of how we implement the analysis taking into account that we have a signed, directed network). We begin discussing the corresponding results by looking at the network at the global level, i.e., including all the students and all their relationships in a single network. Figure 6 represents the global network obtained from the two waves, with the nodes separated in communities. The plotting algorithm is ForceAtlas2^38^, which yields itself a community analysis—as a matter of fact, the high correlation between colors and position in the plot is an outcome of this algorithm and has not been externally imposed. This in turn means that the communities found are quite robust. As can be seen from the data summarized in Table 3, in the network of wave 1 community C1 is formed by the students in the two English-speaking groups, whereas community C3 contains groups C and D and community C4 contains group E; community C2 is formed by a minority of students from almost every group, but mostly from D. The separation observed in the plot between community C1 and the rest speaks of the deep separation between the two English-speaking groups and the three Spanish-speaking ones. This division most likely originates from the fact that students that just entered the high school two months prior to wave 1 still rely more on colleagues from the same primary school. Unfortunately, we do not have data on their school of origin, which could help us understand why the Spanish-speaking groups end up separated in two communities.

**Table 3:** Distribution of the communities by group in wave 1 (left) and wave 2 (right).

	C1	C2	C3	C4
A	27	2	0	0
B	28	3	0	0
C	0	3	27	0
D	0	10	20	0
E	0	0	2	29
Total	55	18	49	29

	C1	C2	C3	
A	28	1	0	
B	31	0	0	
C	0	8	22	
D	2	10	18	
E	0	2	29	
Total	61	21	69	

It is worth noting that the plot of the network in wave 2 looks more spatially extended than that of wave 1. This is a consequence of the almost doubling in the number of bad relationships mentioned above while the number of positive links remains more or less constant. However, at the level of the whole group negative links do not seem to have a strong influence in the community structure. As can be seen from the figures in the Supplementary Information, if the community analysis is carried out without the negative links, the only change is in wave 1, where the community arising from group E is merged with community C2, formed by an assortment of students from all Spanish-speaking groups. In wave 2 there is not any significant change in the number or composition of the clusters. Therefore, we conclude that identity factors (English- vs Spanish-speaking, along with school of origin) are what is most relevant at this level and negative links lead to small changes, if at all.

In wave 2, Fig. 6 shows that even if a whole academic course has passed, the separation between groups speaking different languages still remains. However, the increase in the number of friends is visible in the fact that the two communities are now somewhat closer. Interestingly, our algorithm detects one fewer community, the reason being that now all Spanish-speaking groups appear in a single community, C3. C1 continues to be the English-speaking part of the students, now including all but one of them, while C2 is a smaller community formed by people of groups C and D. It is interesting to note that group D remains in the two waves split into two communities, being the only one that is thus separated. It is very likely that there is something acting as a dichotomizing criterion here, such as the school or the country of origin, but unfortunately we do not have the data to assess the mechanism explaining this splitting. The table of the number of people that change between communities in the Supplementary Information confirms that the movements of individuals between communities took place as we have just discussed. It can also be seen from the numerical data that while communities C1 and C3 have the gender distribution that they inherit from the constituent groups, with a 2:1 girl-to-boy ratio in C1 and almost 1:1 in C3, C2 is practically a male only community, with 19 boys and 2 girls.

**Figure 7:**
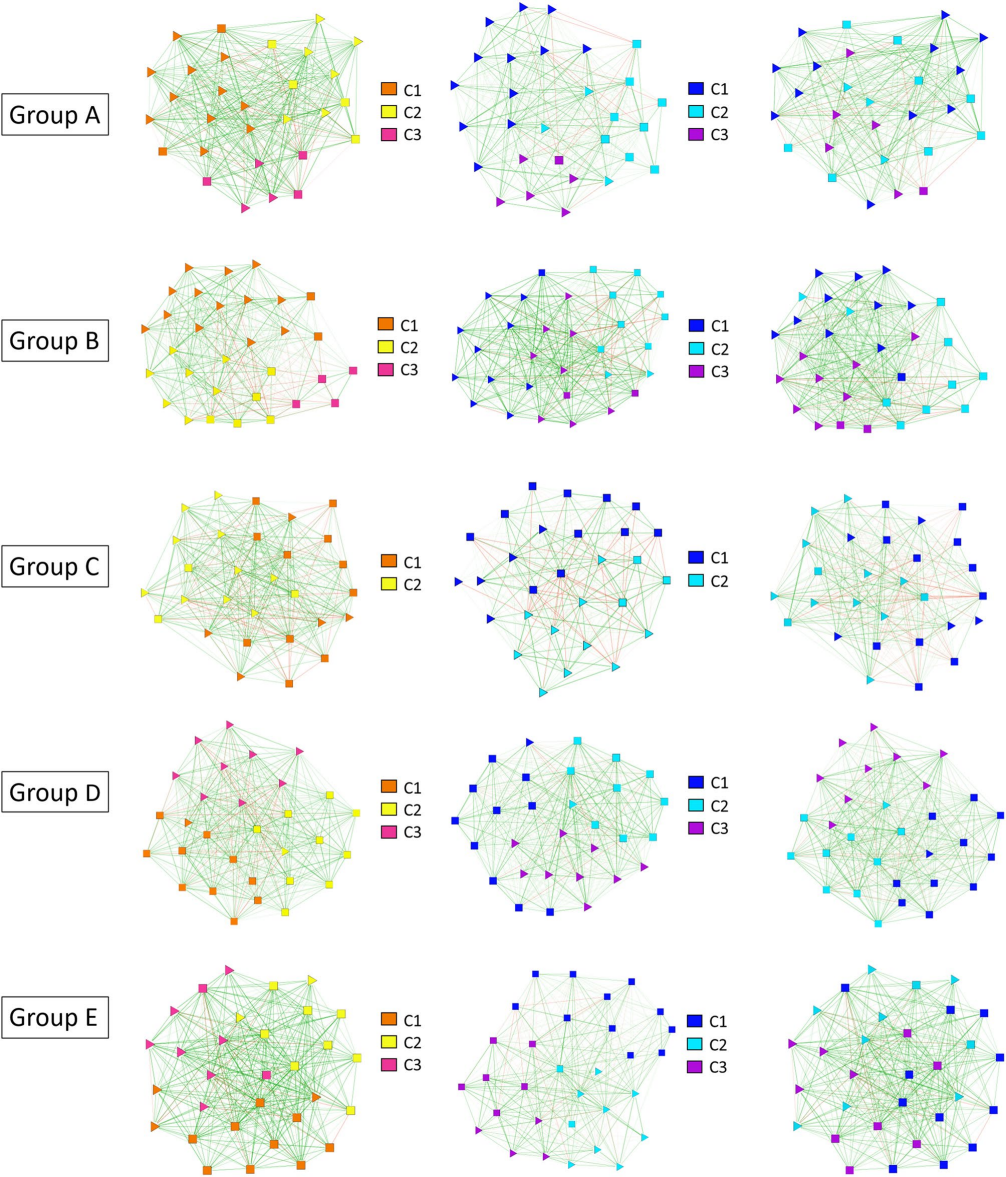
Network representation of all the social relations within each group in wave 1 (left) and wave 2 (center) along with the community structure of the network. The networks on the right also correspond to wave 2 (center) but, in order to facilitate comparison, nodes are laid out in the same position as in wave 1, whereas links and color codes for communities correspond to wave 2. The shape of each node represents its gender: triangles for girls and squares for boys.

Having discussed the network as a whole, we now turn our attention to each group separately. For this study, we consider only the links within the groups and discard the reported friendships with students in other groups. The main feature observed at this level is a strong division by gender. For instance, consider group C (cf. Fig. 7 and tables in the Supplementary Information). The group is formed by 15 boys and 15 girls, and in wave 1 our community algorithm returns two communities, one with 12 boys and 5 girls, and another one with 10 girls and 3 boys. This division remains in wave 2, with only two students exchanging communities. However, when the groups are less gender-balanced, the situation becomes more complicated. Take one of the English-speaking groups, e.g. group A, with 20 girls and 9 boys. In wave 1, we find a community consisting of 11 girls and only 2 boys, whereas the other communities are better (or exactly) gender balanced. In wave 2, we again find three communities but now all of them are gender dominated: 12 girls and 0 boys, 3 girls and 8 boys, and 5 girls and 1 boy. As can be seen from the top panel of Fig. 7, the process has been quite complex, with a lot of students exchanging communities: if we look for instance at community C1 in wave 1, of its 13 original members only 5 remain in it, with two groups of 4 moving to the other two communities. This reinforces our interpretation of an evolution strongly dominated by gender homophily, but also suggests that the limits imposed by Dunbar’s circles are destabilizing the process of forming a unique, large cluster composed only by same gender children, making for a situation that is difficult to accommodate. The same is observed in the other groups, with the exception of group D, which in comparison turns out to be remarkably stable. Its 10 boy-1 girl, 9 boy-1 girl, and 9 girls only clusters almost do not not change between waves (again, with the exception of 3 students who exchange communities). We stress here that our algorithm is agnostic with respect to the number of communities it should produce, finding simply the best partition in terms of modularity (see Methods). Therefore, the fact that we never observe a cluster with more than 13 persons of the same gender in spite of the gender homophily bias, along with the intense dynamics of the communities, support the idea that other mechanisms, such as those behind the formation of Dunbar’s circles, are at work here.

These findings about the community structure must also be contrasted with the different types of evolution of the parameter $\mu$: in groups B and D, where the community structure is more stable, we observe that for a large percentage of the group their value of $\mu$ decreases, indicating that relationships with best friends (circle 1) are becoming more relevant. Conversely, in the other groups $\mu$ increases for practically all the students, in a manner consistent with the exploration of new personal networks and the restructuring of the communities. Thus, it appears that the evolution of $\mu$ might indeed be linked to structural properties of the groups. An important caveat is in order here, namely that Dunbar's circles refer to ego- or personal networks, the set of relationships of one person, whereas communities pertain to the realm of social networks, the set of all relationships among a set of people. Still, we believe that the connection we have suggested above makes sense in view of the fact that, as stated above, our networks are highly reciprocal, turning the clusters into something closer to cliques. In the limit of a perfect clique, everybody’s personal network would contain everybody else; with our percentage of reciprocity, personal networks in a cluster still contain most of the rest of the group and thus the Dunbar limits could have a say in their evolution.

**Figure 8:**
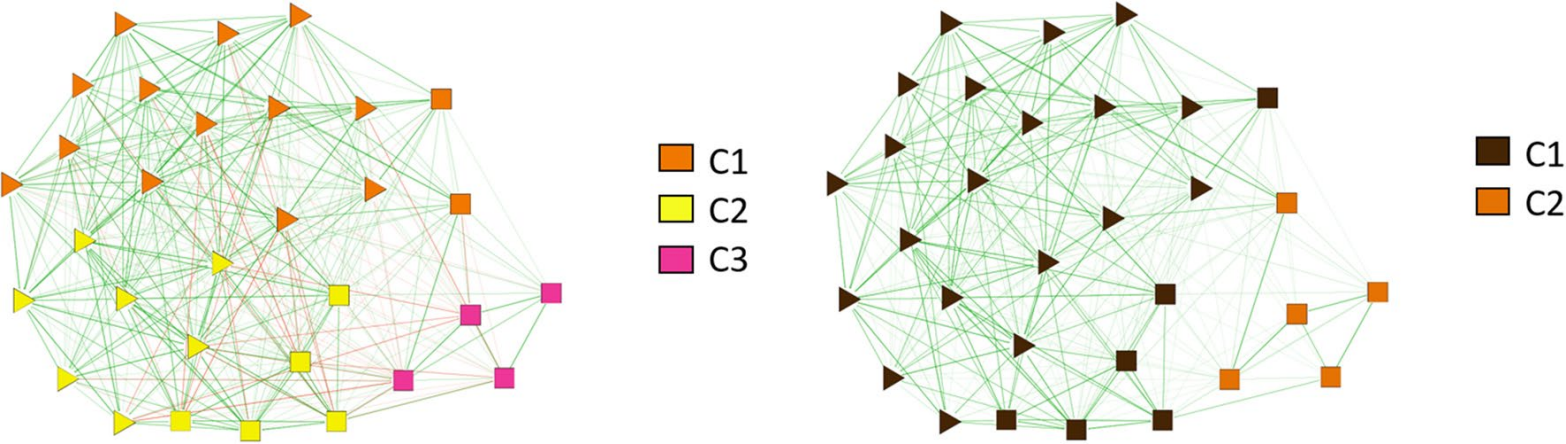
Community analysis of group B in wave 1 taking into account both positive and negative links (left) and only positive links (right). Nodes belonging to each community are marked by color as indicated in the plot. Friendship links are drawn in green, whereas enmity links are drawn in red. The shape of each node represents its gender: triangles correspond with girls and squares with boys.

There is an interesting analysis that reveals the key role played by the negative relationships, despite their relative small number, at the scale of the group. Figure 8 shows an example of the differences in the community structure of the network corresponding to group B in wave 1 with and without the negative links. We can see how communities C1 and C2 would merge together almost perfectly if the negative links did not exist. Given that the resulting community would contain 26 nodes compared to the 15 and 12 of the actual communities, one wonders if the existence of negative links is tied to the natural size of the sympathy network, so that it would be more likely for them to appear when the community exceeds its typical size. Another example of the same phenomenon is shown in the Supplementary Information (notice that in none of the groups or waves the opposite ever happens, i.e., there are never two communities in the network of only positive links that merge into one by inclusion of negative links). We note that contrary to what happens at the level of the whole network, by looking at relationships within single groups the results can not be understood in terms of us vs them or ingroup vs outgroup (or identity labels), thus leading to a more relevant role for the negative links. This suggestive hypothesis deserves further scrutiny.

Finally, a natural question about a network with positive and negative links is whether it satisfies social balance. The theory of social balance^41–44^, captured by the aphorism ‘my friend’s friend is my friend’, posits that triangles with an odd number of negative links create a cognitive dissonance that makes them unstable and prone to be ‘resolved’ into a more balanced configuration (by changing the sign or removing one link). A perfectly balanced network can be decomposed into two positive subnetworks joined by purely negative links^42, 43^. Under this strict definition of balance, no real network with positive and negative links is ever balanced—ours contain many unbalanced triangles. However, one can relax this condition by introducing a null model against which to test whether the number of unbalanced triangles can be considered relevant or not. The usual null model to test social balance in a network is obtained by randomly permuting the links belonging to some triangle without changing their signs. This test has been criticized though^45^, on the basis that positive and negative networks have very different properties—while the latter are essentially random, the former have structure. Thus, permuting both kinds of links can alter the nature of the network and overestimate imbalance. A less biased null model can be obtained if links are classified according to their embedding (number of triangles which they belong to) and only links with the same embedding are permuted^45^.

**Figure 9:**
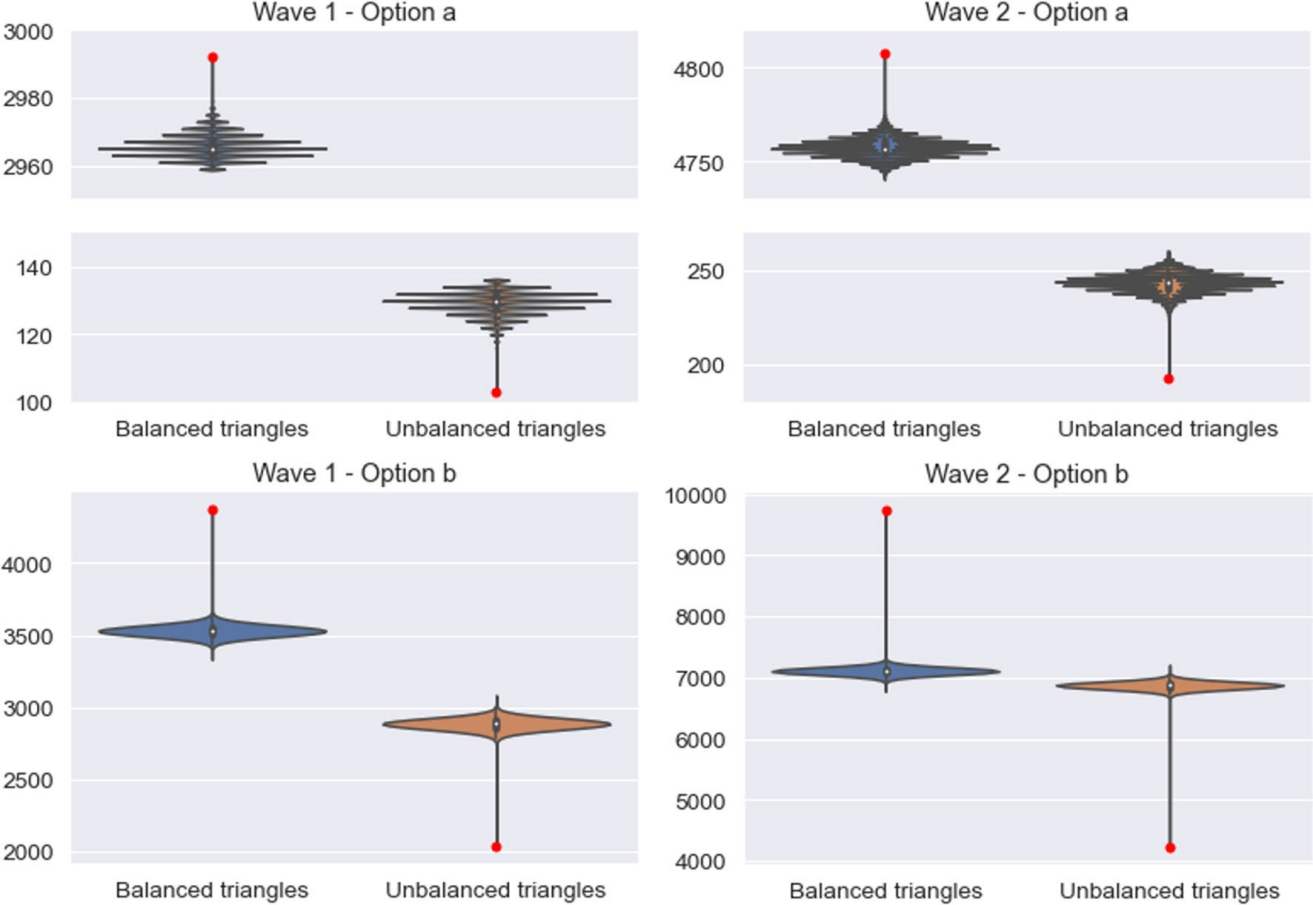
Comparison between the observed number of balanced and unbalanced triangles and the results obtained in the simulations. The red point represents the observed value and the violins correspond to the simulations. Left: Results obtained for the wave 1. Right: same for wave 2. Top: Option a: a link is considered negative only if the interaction between a pair of nodes is negative in both directions. Bottom: Option b: a link is considered negative as soon as there is a single negative interaction between a pair of nodes.

Our networks contain links of two intensities, which calls for an extension of the theory of social balance. However, negative links are too few to maintain such a distinction and still have statistical significance. Accordingly, for testing social balance we have ignored the intensity of links. Also, our links are directional and not always reciprocated. Even if they are, often the signs of the two directional links are different. We do not know why this happens, but the number of opposite-sign directional links is not negligible. In view of this, we have adopted two criteria to project our network onto a bidirectional network: (a) consider a negative link only if the interaction between a pair of nodes is negative in both directions, or (b) assuming that the presence of a single negative interaction between a pair of nodes is already an evidence of a conflict and also consider those cases as negative links. The number of triangles with 0 through 3 negative links is very different in both cases (see Table S11 of the Supplementary Information), but regardless of this, embedding-preserving permutations never produce configurations with fewer unbalanced triangles than the empirical network—as a matter of fact, except for very few cases, this number is actually larger (see Fig. 9). We then conclude that, even though the existence of negative links creates lots of unbalanced triangles, the configuration in which they appear is compatible with a very strong bias toward balance.

## Discussion

In this paper, we have studied the evolution of positive and negative relationships among students in a high school by looking at two waves of data collection separated by several months. In both surveys, we find evidence for a circle structure for positive friendships, similar to the one proposed by Dunbar, although the typical numbers for Dunbar’s circles are only found when looking at intra-group relationships. We suggest that this may come from the fact that students think differently of their intra-group and their inter-group relations. Importantly, and beyond the question of the circle sizes, our results show that when students have just arrived at the high school, their relationships are intense and with a limited number of classmates, i.e., they have a structure which resembles cliques involving typically 4–6 students. By the end of their first year in high school, the structure of their relationships has experienced a large change, with fewer intense friendships and more friendships in general, becoming more resemblant of a circle distribution. We have been able to precisely quantify this evolution by means of a parameter, $\mu$, that is obtained by a fit to the students’ reported answer. We find a very small number of reported negative relationships, which nevertheless increases from the first to the second wave.

As a way to gain further insight on the evolution of relationships, we have looked at the community structure of the whole social network both at the level of the five groups considered together and at the group level. The communities found from the analysis of the first wave are somewhat mixed, made up of both boys and girls, but they evolve so that in the second wave they appear to be largely segregated by gender. At the group level, the size of each community was stabilized around 12 people, which coincides with the size of the second Dunbar’s circle, known as sympathy group in social psychology. As a consequence, in classes with around 20 students of the same gender, their group split in two separate communities of about 10 each to stay below the second Dunbar’s circle. Interestingly, we have also observed that for this separation to arise in the community analysis it is necessary to include the negative relationships; otherwise, the analysis yields a unique community formed by almost all students of the same gender. Another relevant finding is that when the community structure is more stable, relationships with best friends intensify, and there is an evolution opposite to the general one of cliques relaxing to circles: more stable communities result in a decrease of the $\mu$ parameter and in an intensification of the focus on best friends (circle 1).

The existence of negative links puts the question of balance into play. The theory of social balance predicts that triangles with an odd number of negative links create a cognitive dissonance that renders them unstable and prone to disappear over time. Ideally, social networks should be free of unbalanced triangles. However, negative links are constantly being formed, so the question is not so much whether the network is free from those triangles as to whether they appear in a number that is significantly below what a null model would yield for that particular number of negative ties. We have run a recently proposed test whose null model takes into account the different nature of the positive and negative subnetworks, and have found that in one million realizations of the null model a network with fewer unbalanced triangles than the original network never shows up. This result forces us to conclude that there seems to be a strong tendency to reduced imbalance in middle-school classrooms.

Aside from their relevance towards understanding how we organize our relationships and how they evolve in time, which is particularly important in such a crucial age in development as early adolescence, we believe our results have practical implications about the school social network and atmosphere. Thus, we have observed that the co-existence of two working languages in the same high school leads to the splitting of the social network of the school in two groups with very little communication or very few positive connections. To avoid this, it would be important that the school direction would design some joint activities so that the students of both groups would get to know each other. Another very relevant finding is the instability associated to gender imbalance in the groups. In such a situation, the gender homophily typical of this age leads to tensions between all students of the majority gender wanting to be in the same community and the fact that there are limits to the number of friendships of a given intensity. In this case, we have seen that negative relationships appear that split the majority gender community in two, which could lead to increased polarization of the group atmosphere. Our results suggest that this is a problem that can be easily avoided by making gender balance a priority or else by reducing the group size (which may not be possible, like in public schools in the Madrid region whose group size is determined by the Regional Government). Another related measure would be to take this into account when forming groups in subsequent academic years, by separating students belonging to such gender-segregated communities in different classes to avoid perpetuating intra-group divisions. All these insights point to the fact that, beyond its very appealing interest from the scientific viewpoint, this kind of social network analysis is an efficient and easy to implement tool that can be used to foster a friendlier school environment which could also have connections with students’ performance.

## Methods

This study was approved by the Ethics Committee of Universidad Carlos III de Madrid, the institution responsible for funding the project, and the surveys were subsequently carried out in accordance with the approved guidelines. In particular, informed consent was obtained from both the participating students and their parents or legal tutors.

### Exploring social structure: the $\mu$ parameter

We explored the social structure for each individual following the model presented in Ref.^20^, which explains Dunbar’s circle structure as a result of allocating a finite cognitive resource among a certain number of links. According to the model, if layer *k* has $l_k$ links and an associated cost $s_k$, for each individual, the ratio of the number of links between consecutive layers is given by $l_{k+1}/l_{k}=e^{\mu \left| \Delta s_k\right| }$, where $\left| \Delta s_k\right| =\left| s_{k+1}-s_k\right|$ is the costs increment between consecutive. The assumption $|\Delta s_k|=1$ explains the constant proportionality between successive layers.^20^ We have considered only two intensity levels of relationship: $k=1$ (‘best friend’ for positive and ‘worst enemy’ for negative relationships), and $k=2$ (‘friend’ for positive and ‘enemy’ for negative relationships). Thus

1 $$\begin{aligned} \mu =\log \left( \frac{l_2}{l_1}\right) =\log \left( \frac{C_2-C_1}{C_1}\right) , \end{aligned}$$

$C_i$ being the number of links in clircle *i*. When a participant selects another student, a directed, weighted link is created. Its weight can be $-2,-1,1,2$ depending on whether the reported relationship is “worst enemy”, “enemy”, “friend”, “best friend”, respectively. In a few cases the same relation is reported simultaneously as “friend” and “best friend” or as “enemy” or “worst enemy”. In those cases the weight corresponds to the most intense one. In other few cases the relation is marked both, as positive and negative. To resolve this we have added the corresponding weights, removing the link if the resulting weight is zero. Once each link has a well-defined weight, the calculation of $\mu$ for positive ($\mu ^+$) and negative ($\mu ^-$) relationships is achieved using equation 1.

### Community detection

To solve the problem of community detection we use the modularity optimization method proposed by Newman ^46^, which allows us to determine how far is the studied network structure from a random distribution of the links. More specifically, in order to incorporate all the information collected from the student surveys, we make use of the most general definition of modularity *Q*, which considers the existence of directed, weighted and signed links ^47^. That is

2 $$\begin{aligned} Q=\frac{w^{+}}{w^{+}+|w^{-}|} Q^{+}-\frac{|w^{-}|}{w^{+}+|w^{-}|} Q^{-}, \end{aligned}$$

where $w^+$ and $w^-$ represent the total strength of positive and negative relationships, respectively. The construction of *Q* using both the positive ($Q^+$) and negative ($Q^-$) modularities implicitly involve the possibility of establishing signed links independently. Therefore, the total modularity represents the equilibrium between the inclination of positive relationships to build communities and the tendency of negative ones to break up them. For our particular case of study, links in the network can only take values $\left\{ -2,-1,1,2 \right\}$ depending on the relationship sign (enmity or friendship) and intensity.

The positive modularity $Q^+$ is defined as

3 $$\begin{aligned} Q^{+}=\frac{1}{2 w^{+}} \sum _{i,j}\left( w_{i j}^{+}-\frac{w_{i}^{+,out} w_{j}^{+,in}}{2 w^{+}}\right) \delta \left( C_{i}, C_{j}\right) , \end{aligned}$$

where $w_{ij}^+$ is the (*i*, *j*) element of the positive weighted adjacency matrix and $w_{i}^{+,out}$ and $w_{j}^{+,in}$ are the outer and inner positive strengths associated to each node. $C_{i}$ represents the community which individual *i* belongs to, so the Kronecker delta $\delta (C_i,C_j)$ takes value 1 if both nodes share community and 0 otherwise. The negative modularity $Q^-$ is defined by an analogous expression to 3 but using the absolute values of the negative weights.

The optimization of modularity is a NP-hard problem, due to the number of possible combinations growing exponentially with the number of individuals. For this reason, we use a combination of heuristic algorithms (routines *extremal, louvain, reposition, spectral, tabu,* and *fast* from the software package *Radatools,* https://deim.urv.cat/~sergio.gomez/radatools.php) to find the best partition of the network into communities (the one which maximizes *Q* in equation 2).

## Supplementary Information

Supplementary 1.
