# Supplementary material for: Evolution of social relationships between first-year students at middle school: from cliques to circles
Source: Sci Rep. 2021 Jun 3;11:11694. doi: 10.1038/s41598-021-90984-z (PMC8175354; doi:10.1038/s41598-021-90984-z)
Supplement: Supplementary file 1 — Supplementary 1. [file 41598_2021_90984_MOESM1_ESM.pdf]

Evolution of social relationships between first-year students at middle school: from cliques to circles

Diego Escribano,[1,2] Victoria Doldán-Martelli,[1,2] Francisco J. Lapuente,[3,4]
José A. Cuesta,[1,2,5,6] Angel Sánchez[1,2,5,6]

[1]Grupo Interdisciplinar de Sistemas Complejos, Departamento de Matemáticas, Universidad Carlos III de Madrid, 28911 Leganés, Madrid, Spain

[2]Unidad Mixta Interdisciplinar de Comportamiento y Unidad Social (UMICSS) UC3M-UV-UZ, Universidad Carlos III de Madrid, 28911 Leganés, Spain

[3]Instituto de Enseñanza Secundaria Blas de Otero, 28024 Madrid

[4]Departamento de Biología y Geología, Física y Química Inorgánica, Universidad Rey Juan Carlos, 28933 Móstoles, Madrid

[5]Institute for Biocomputation and Physics of Complex Systems (BIFI), University of Zaragoza, 50018 Zaragoza, Spain

[6]UC3M-Santander Big Data Institute (IBiDat), 28903 Getafe, Spain

Supplementary information

Survey data

Table S1 presents the statistics of the students.

	**Boys**	**Girls**	**Total**
**A**	9	20	29
**B**	11	20	31
**C**	15	15	30
**D**	19	11	30
**E**	19	12	31
**Total**	73	78	151

Table S1: Distribution of the students by group and gender. It is valid for both December 2018 and May 2019 data.

Table S2 presents the results on the percentage of reciprocal relations both within the whole network and group by group. For comparison, values of reciprocity obtained from randomly rewiring the network arising from the survey 1000 times are given, along with confidence intervales. Table S3 reports the same for wave 2.

Group	Observed	Simulated
**Complete**	0.566	0.226 (0.214; 0.238)
**A**	0.639	0.556 (0.528; 0.584)
**B**	0.783	0.568 (0.534; 0.602)
**C**	0.596	0.433 (0.395; 0.471)
**D**	0.795	0.700 (0.678; 0.722)
**E**	0.610	0.497 (0.467; 0.527)

Table S2: Reciprocity of positive links in the first wave. The observed column corresponds to the value of the real network. The simulated column corresponds to the mean value obtained after 1000 simulations rewiring the links. The values in parenthesis are the mean value minus/plus two times the standard deviation.

Group	Observed	Simulated
**Complete**	0.582	0.246 (0.234; 0.258)
**A**	0.688	0.597 (0.574; 0.620)
**B**	0.827	0.713 (0.686; 0.740)
**C**	0.625	0.504 (0.468; 0.540)
**D**	0.789	0.694 (0.675; 0.713)
**E**	0.631	0.455 (0.423; 0.487)

Table S3: Reciprocity of positive links in the second wave. The observed column corresponds to the value of the real network. The simulated column corresponds to the mean value obtained after 1000 simulations rewiring the links. The values in parenthesis are the mean value minus/plus two times the standard deviation.

Individual results and Dunbar circles

As indicated in the main text, most students experience an increase in the value of the parameter μ obtained from the fits to the theoretical predictions of Ref. 1. Figure S5 below presents a histogram of the number of students with increasing or decreasing μ as a function of their groups. Figure S8 collects all the differences in μ between waves in the form of a violinplot, to depict the distribution of changes in more detail.

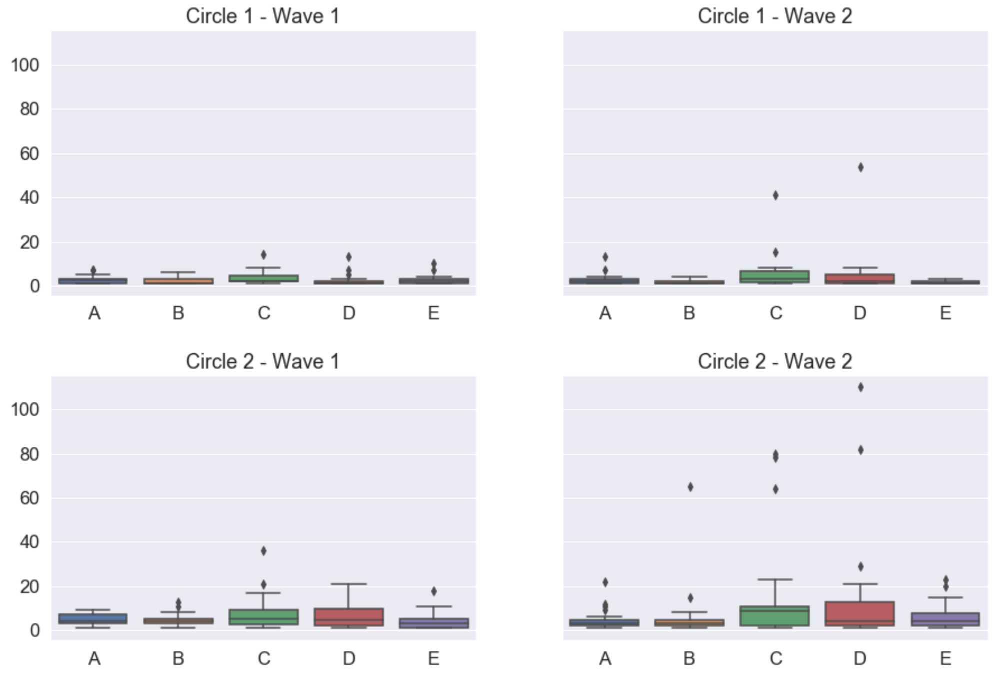

Figure S1: Boxplot of the answers about enemies and worst enemies. Top, numbers of students marked as "worst enemies" by students of each group (circle 1). Bottom, numbers of students marked as "enemies" by students of each group (circle 2). Left, results from wave 1. Right, results from wave 2. Line represents the median, boxes the first and third quartiles, lines, the 95% confidence interval, and dots, outliers

.

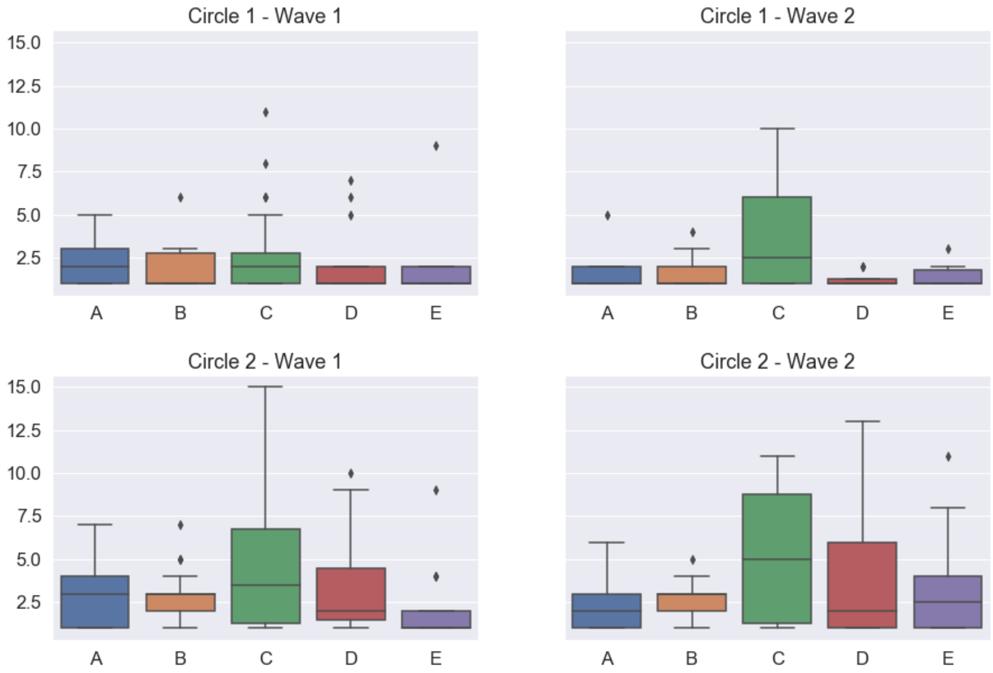

Figure S2: Boxplot of the answers about enemies and worst enemies, restricted to the ego's group. Top, numbers of students marked as "worst enemies" by students of each group (circle 1). Bottom, numbers of students marked as "enemies" by students of each group (circle 2). Only relationships within the group are shown. Left, results from wave 1. Right, results from wave 2. Line represents the median, boxes the first and third quartiles, lines, the 95% confidence interval, and dots, outliers

.

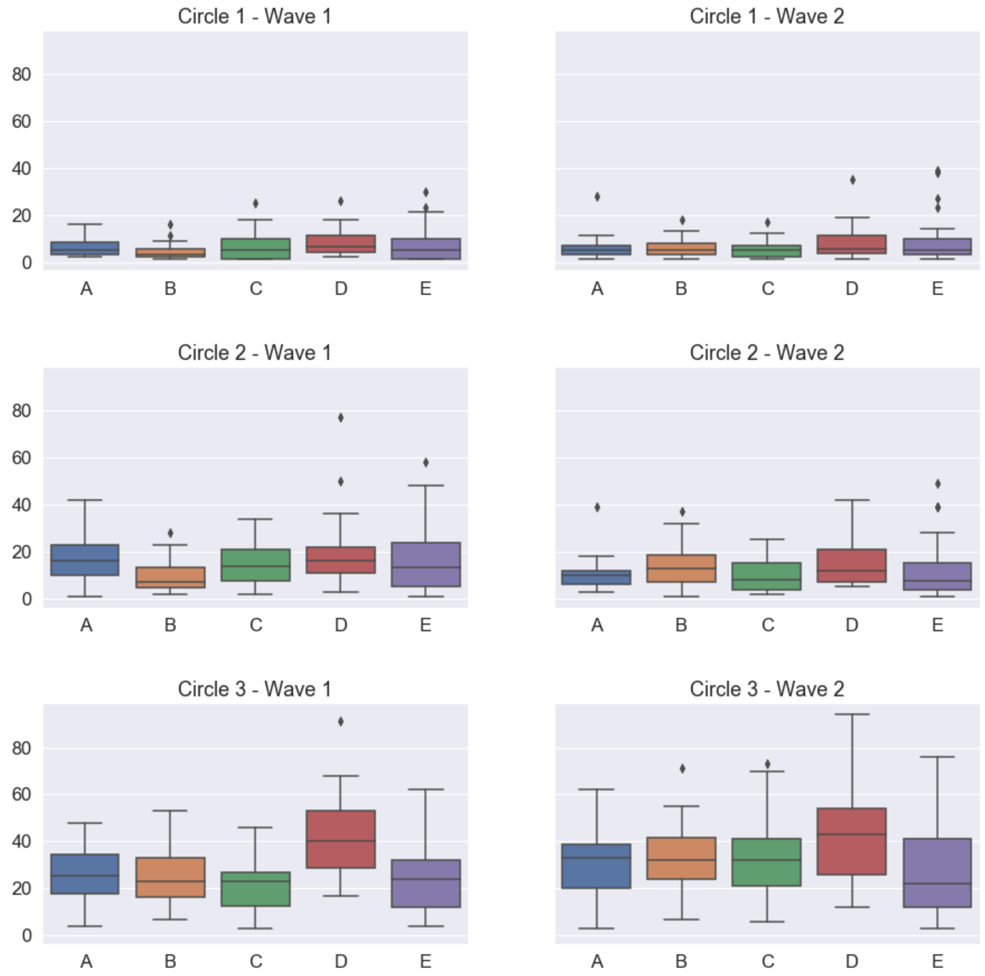

Figure S3: Boxplot of the original answers for the 3 intensity levels of positive relationships prior to merging categories. First row, numbers of students marked as "flesh and bone" by students of each group (circle 1). Second row, numbers of students marked as "close friend" by students of each group (circle 2). Third row, numbers of students marked as "friend" by students of each group (circle 3). Left, results from wave 1. Right, results from wave 2. Line represents the median, boxes the first and third quartiles, lines, the 95% confidence interval, and dots, outliers

.

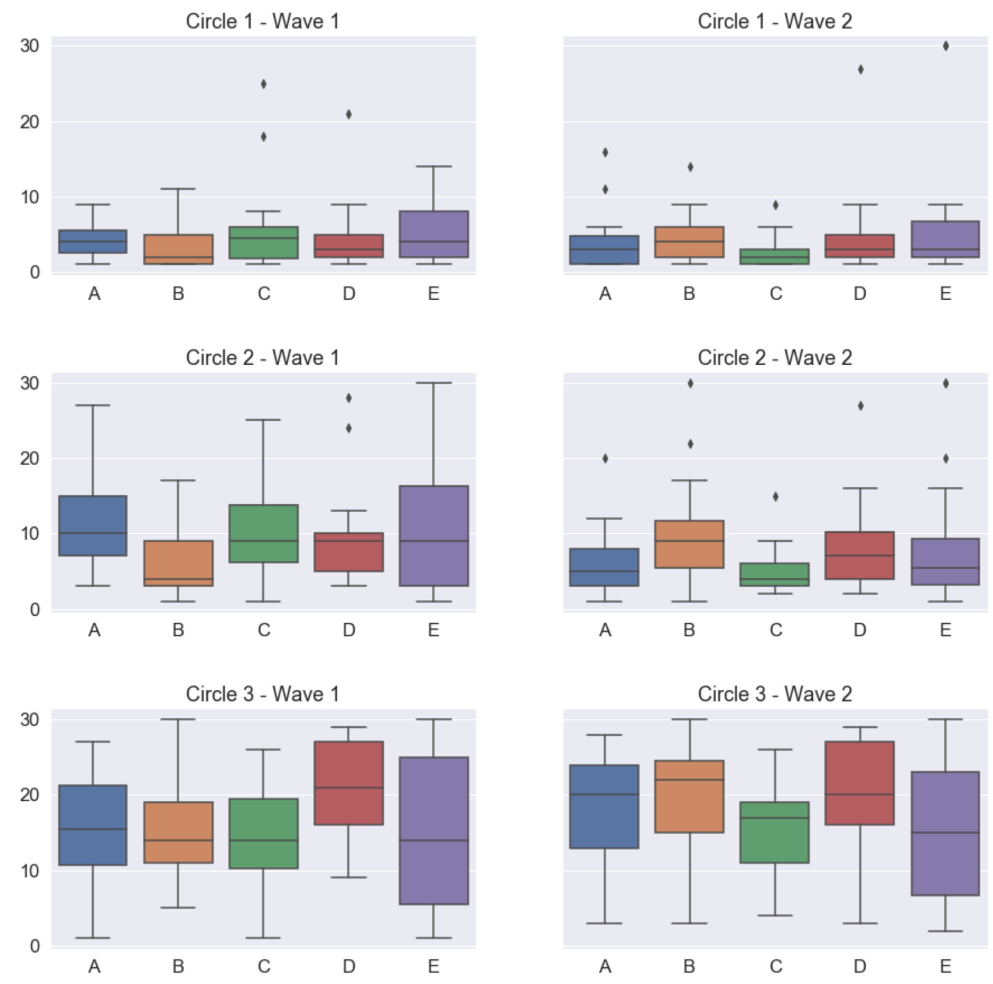

Figure S4: Boxplot of the original answers for the 3 intensity levels of positive relationships prior to merging categories, restricted to the ego's group. First row, numbers of students marked as "flesh and bone" by students of each group (circle 1). Second row, numbers of students marked as "close friend" by students of each group (circle 2). Third row, numbers of students marked as "friend" by students of each group (circle 3). Only relationships within the group are shown. Left, results from wave 1. Right, results from wave 2. Line represents the median, boxes the first and third quartiles, lines, the 95% confidence interval, and dots, outliers

.

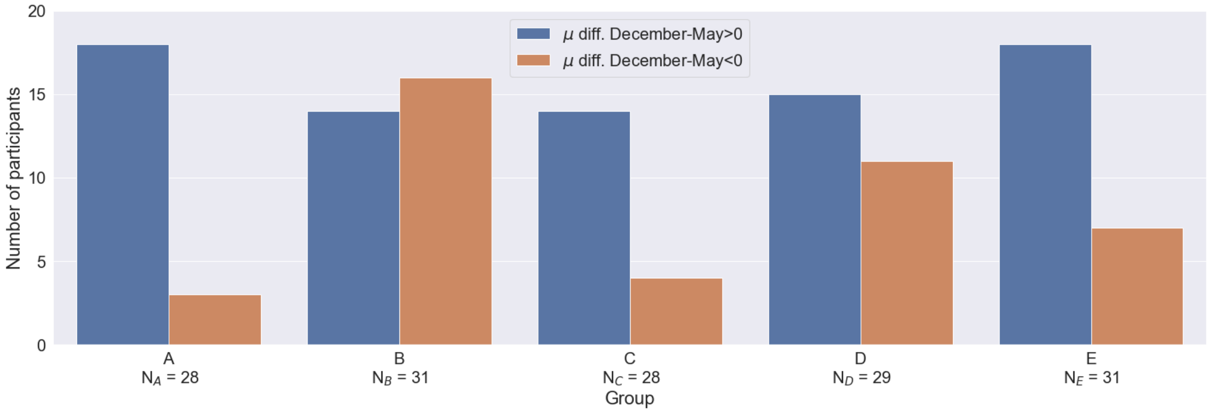

Figure S5: Number of students with increasing or decreasing μ vs group.

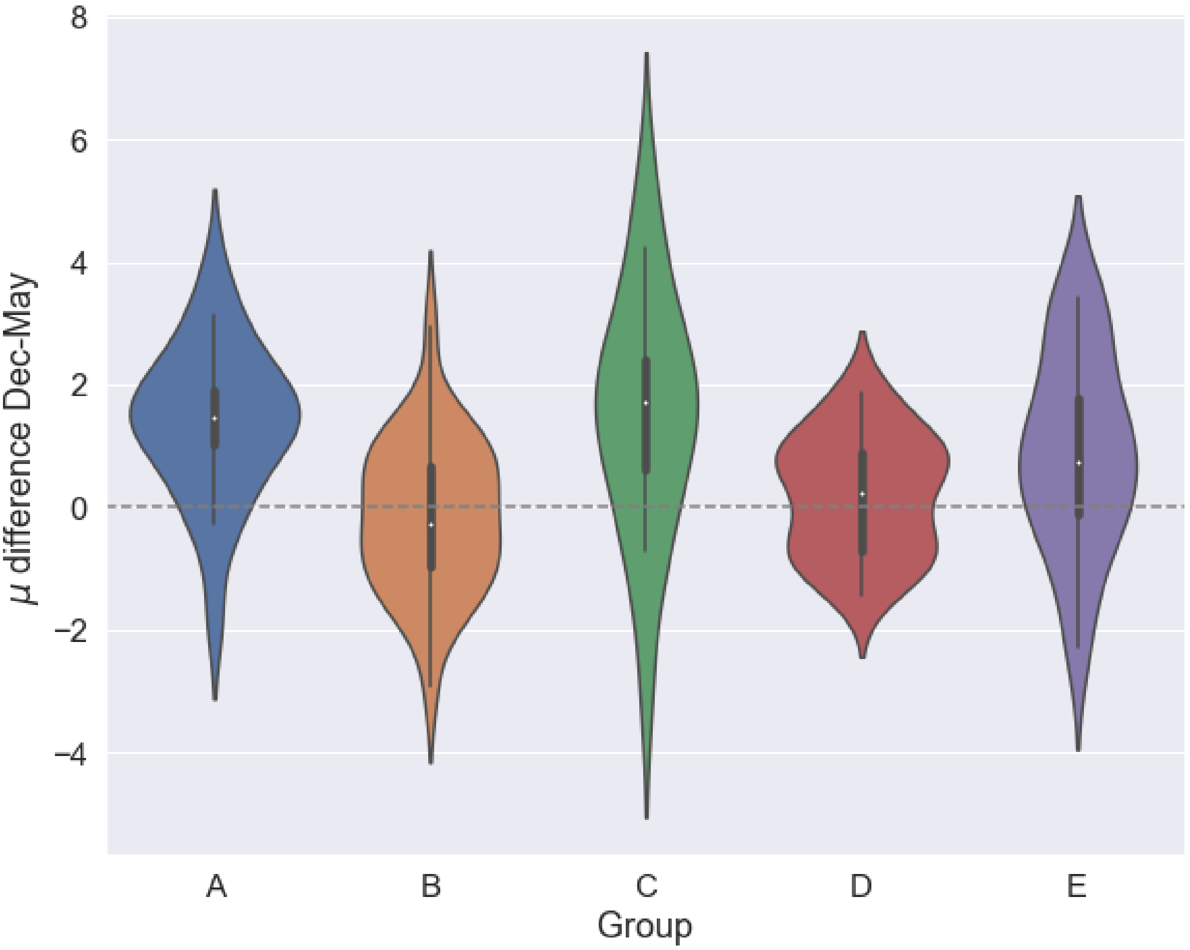

Figure S6: Violinplot of the difference in the μ value between December 2018 and May 2019, for each group. The difference of μ is normally distributed for all groups (Shapiro-Wilk test).

The following figures deal with the ρ parameter, calculated for the number of connections a participant receives, as opposed to the μ parameter, calculated with the number of connections coming from a participant.

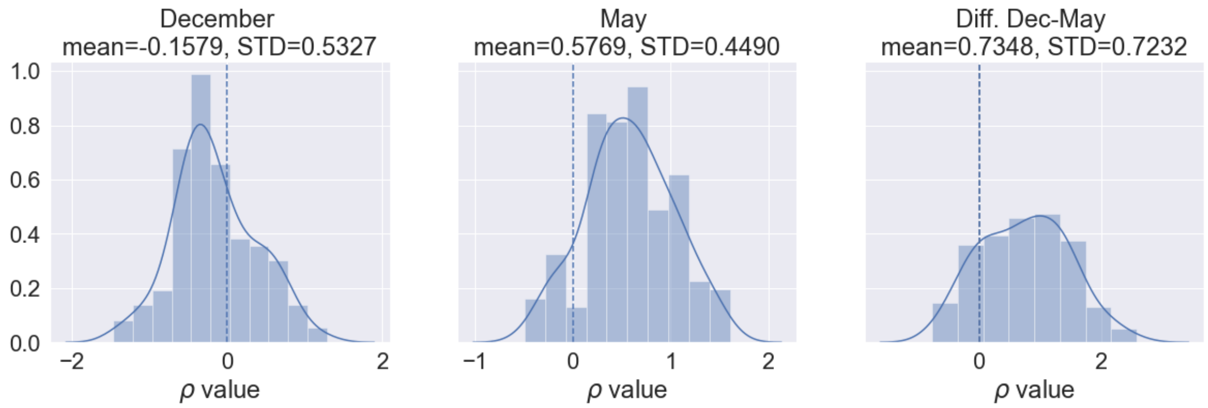

Figure S7: Distribution of ρ values for all the participants in (a) December, (b) May and (c) difference in the ρ value between December 2018 and May 2019. Data distribution fitted using a Kernel Density Estimation (KDE). Vertical dotted line shows ρ=0.

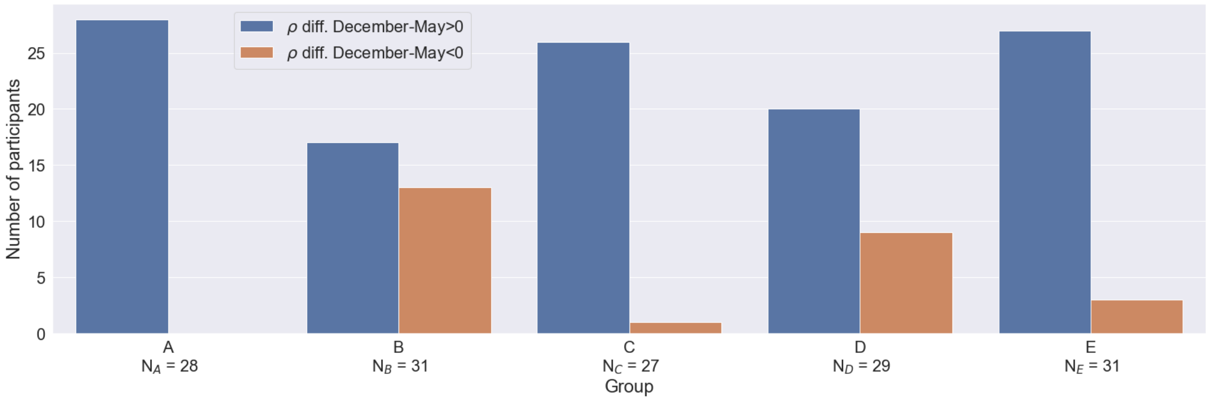

Figure S8: Count plot of the tendency of ρ to increase (ρ difference>0) or decrease (ρ difference<0)during the period of December 2018- May 2019, for each group. The number of observations in which ρ decreases is lower for groups A and C. The size of each group is indicated under the x-axis label.

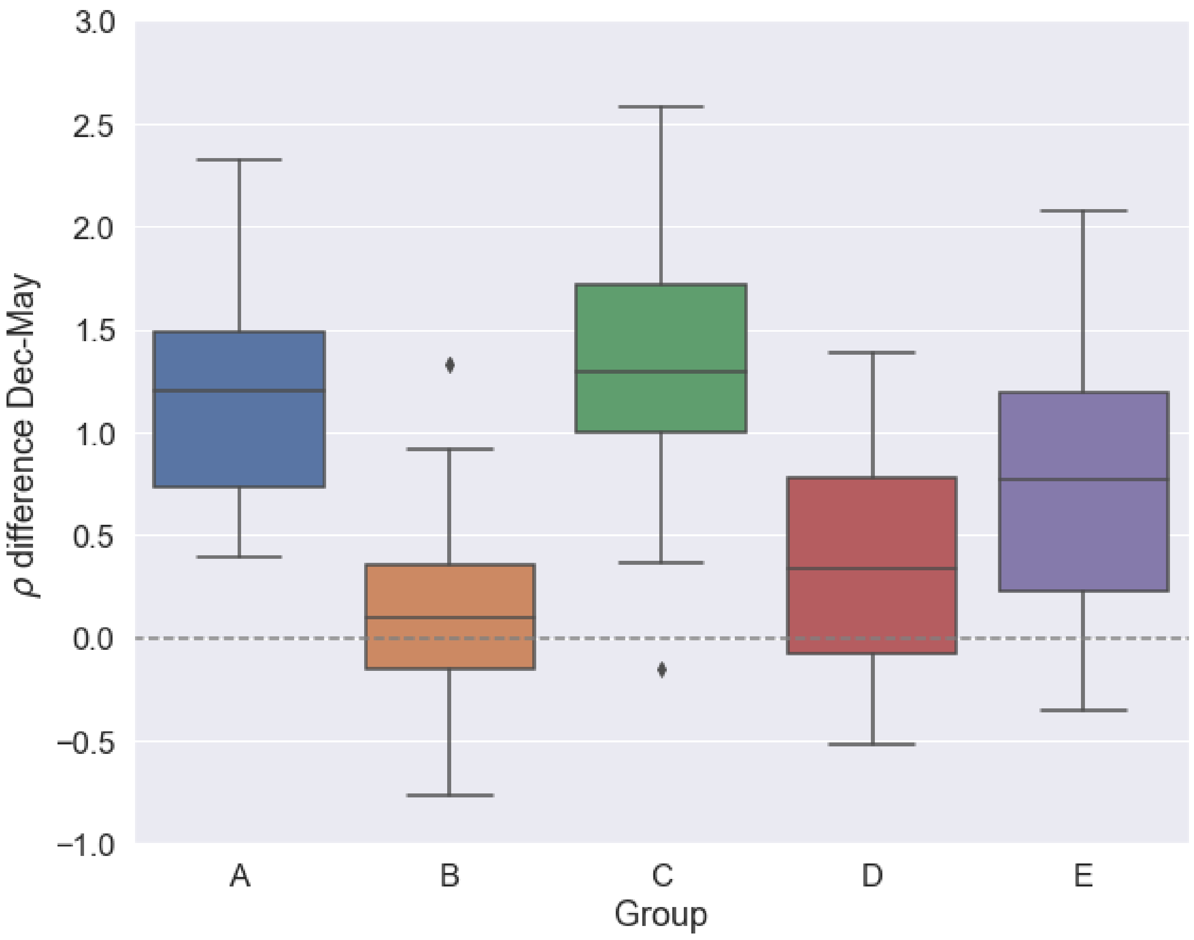

Figure S9: Boxplot of the difference in the ρ value between December 2018 and May 2019, for each group. The difference of ρ is normally distributed for all groups (Shapiro-Wilk test). Groups A and C show a higher increase in ρ.

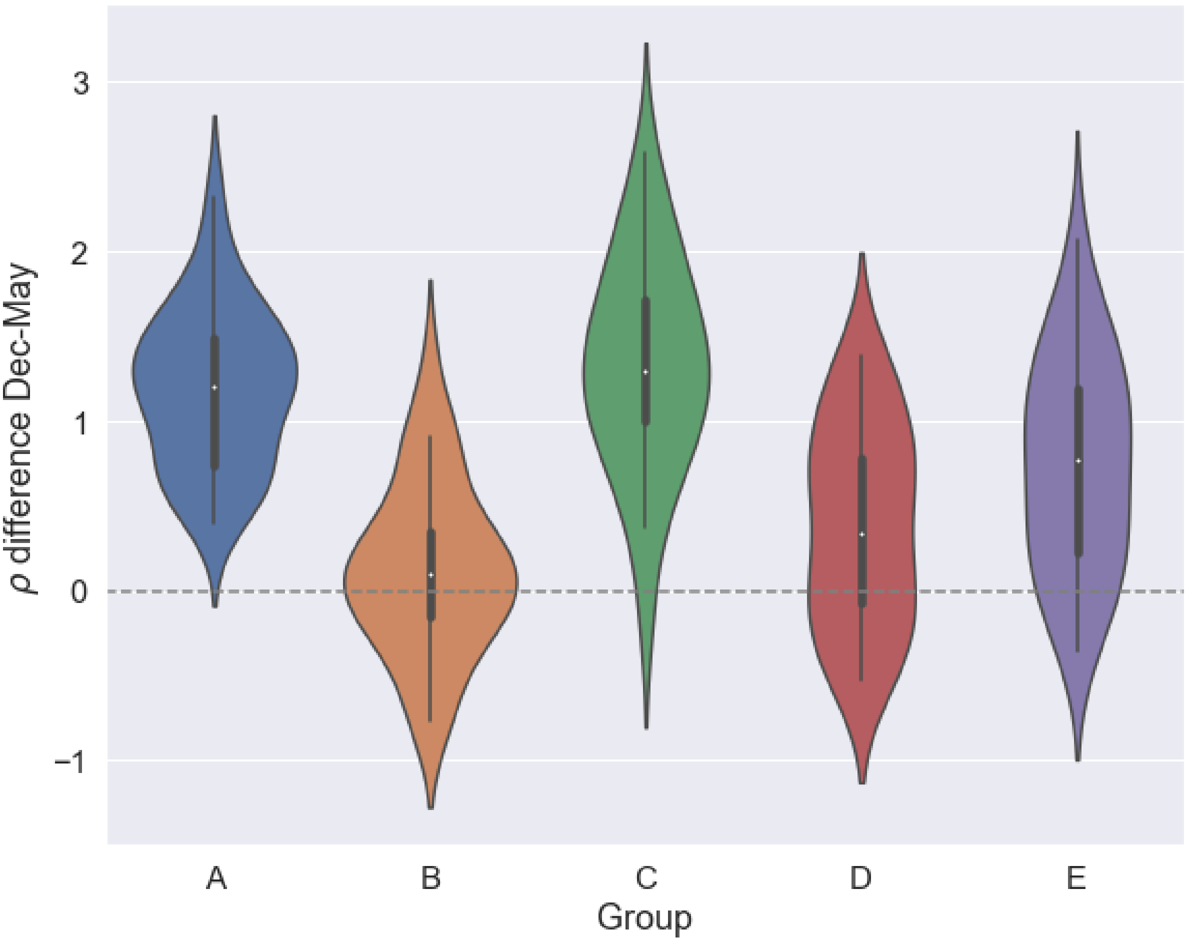

Figure S10: Violinplot of the difference in the ρ value between December 2018 and May 2019, for each group. The difference of ρ is normally distributed for all groups (Shapiro-Wilk test).

Group	Mean (SD) wave 1	Mean (SD) wave 2	Mean (SD) difference
A	−0.431 (0.276)	0.766 (0.345)	1.197 (0.466)
B	0.187 (0.485)	0.315 (0.367)	0.127 (0.501)
C	−0.532 (0.382)	0.789 (0.431)	1.321 (0.628)
D	0.311 (0.438)	0.696 (0.410)	0.386 (0.595)
E	−0.369 (0.430)	0.372 (0.460)	0.741 (0.634)
TOTAL	−0.158 (0.533)	0.577 (0.449)	0.735 (0.723)

Table S4: Mean and standard deviation values for the difference of ρ calculated for the timepoints December 2018 and May 2019.

Network structure and communities

In this section we collect additional information on the network structure of the whole course and of the different groups, as well as on the community structure of the network.

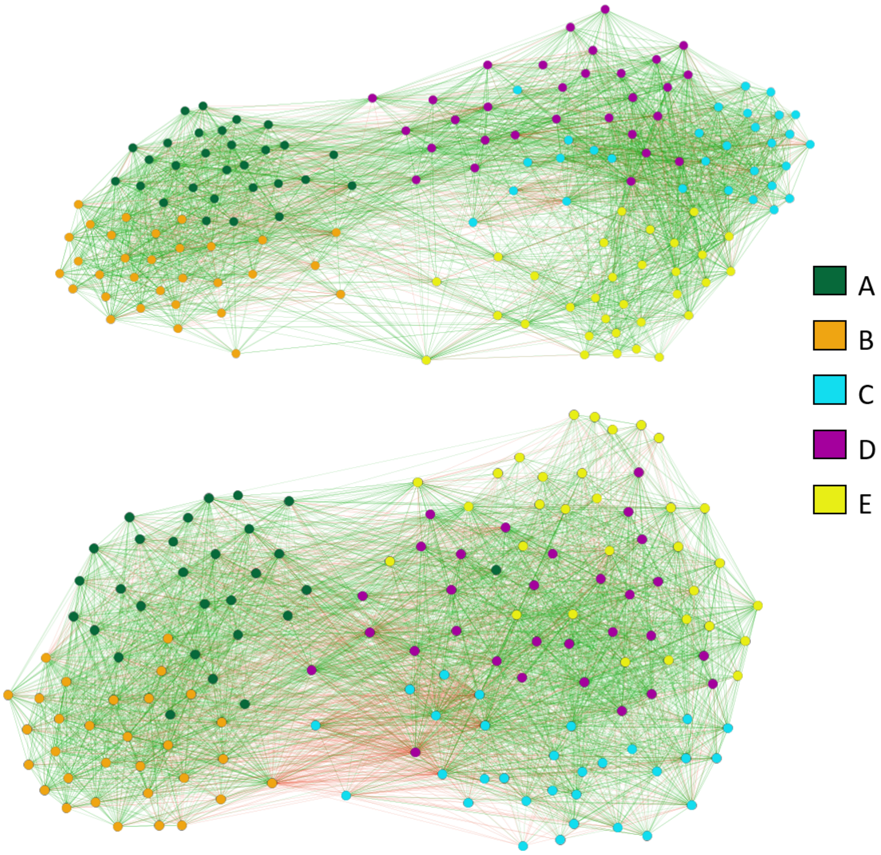

Figure S11: Network representation of all the social relations in wave 1 (top) and wave 2 (bottom) with division by group.

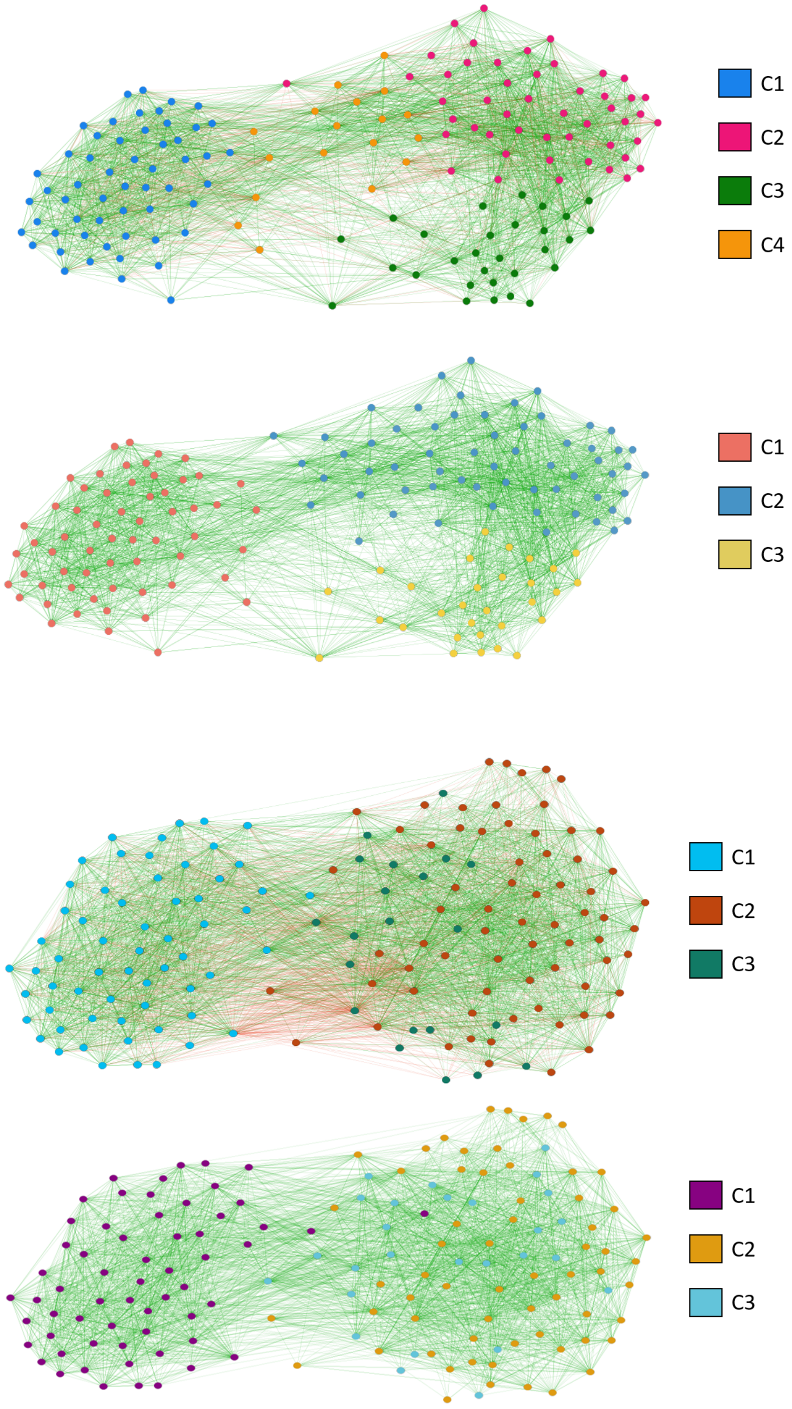

Figure S12: Comparison of community analysis results when considering only positive links and all links. From top to bottom, all links in wave 1, only positive links in wave 1, all links in wave 2 and only positive links in wave 2.

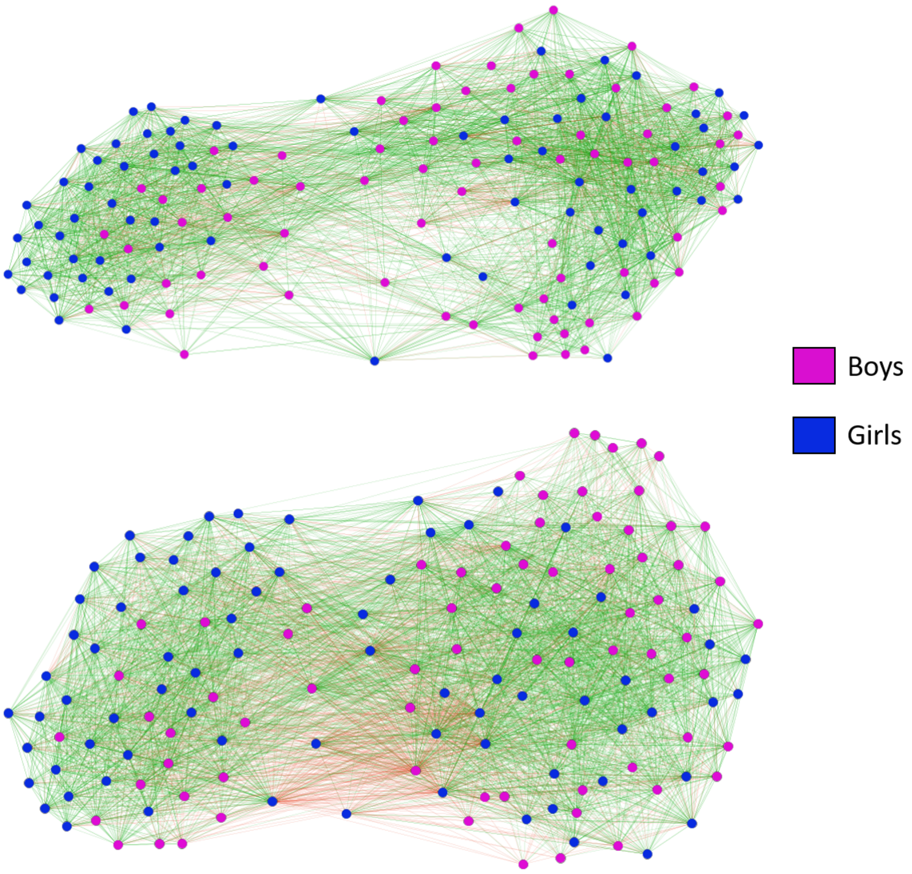

Figure S13: Network representation of all the social relationships in wave 1 (top) and wave 2 (bottom) with division by gender.

	**C1**	**C2**	**C3**	**C4**
**Boys**	15	16	23	19
**Girls**	40	2	26	10
**Total**	55	18	49	29

	**C1**	**C2**	**C3**
**Boys**	20	19	34
**Girls**	41	2	35
**Total**	61	21	69

		**May**			
		**C1**	**C2**	**C3**	**Total**
**December**	**C1**	55	0	0	55
	**C2**	5	12	1	18
	**C3**	1	7	41	49
	**C4**	0	2	27	29
	**Total**	61	21	69	

Table S5: Distribution of the communities by gender globally in wave 1 (left) and wave 2 (center) and transitions between communties (right).

	**C1**	**C2**	**C3**
**Boys**	2	4	3
**Girls**	11	6	3
**Total**	13	10	6

	**C1**	**C2**	**C3**
**Boys**	0	8	1
**Girls**	12	3	5
**Total**	12	11	6

		**May**			
		**C1**	**C2**	**C3**	**Total**
**December**	**C1**	5	4	4	13
	**C2**	6	4	0	10
	**C3**	1	3	2	6
	**Total**	12	11	6	

Table S6: Distribution of the communities by gender in group A in wave 1 (left) and wave 2 (center) and transitions between communties (right).

	C1	C2	C3
**Boys**	2	5	4
**Girls**	13	7	0
**Total**	15	12	4

	C1	C2	C3
**Boys**	1	8	2
**Girls**	10	2	8
**Total**	11	10	10

		May			
		**C1**	**C2**	**C3**	**Total**
	**C1**	10	4	1	15
**December**	**C2**	1	2	9	12
	**C3**	0	4	0	4
	**Total**	11	10	10	

Table S7: Distribution of the communities by gender in group B in wave 1 (left) and wave 2 (center) and transitions between communties (right).

	C1	C2
**Boys**	12	3
**Girls**	5	10
**Total**	17	13

	C1	C2
**Boys**	12	3
**Girls**	5	10
**Total**	17	13

		May		
		**C1**	**C2**	**Total**
	**C1**	16	1	17
**December**	**C2**	1	12	13
	**Total**	17	13	

Table S8: Distribution of the communities by gender in group C in wave 1 (left) and wave 2 (center) and transitions between communties (right).

	C1	C2	C3
**Boys**	10	9	0
**Girls**	1	1	9
**Total**	11	10	9

	C1	C2	C3
**Boys**	10	9	0
**Girls**	1	1	9
**Total**	11	10	9

		May			
		**C1**	**C2**	**C3**	**Total**
**December**	**C1**	2	9	0	11
	**C2**	8	1	1	10
	**C3**	1	0	8	9
	**Total**	11	10	9	

Table S9: Distribution of the communities by gender in group D in wave 1 (top left) and wave 2 (top right) and transitions between communities (bottom).

	C1	C2	C3
**Boys**	9	8	2
**Girls**	3	2	7
**Total**	12	10	9

	C1	C2	C3
**Boys**	11	2	6
**Girls**	0	8	4
**Total**	11	10	10

		May			
		**C1**	**C2**	**C3**	**Total**
**December**	**C1**	5	2	5	12
	**C2**	4	4	2	10
	**C3**	2	4	3	9
	**Total**	11	10	10	

Table S10: Distribution of the communities by gender in group E in wave 1 (top left) and wave 2 (top right) and transitions between communities (bottom).

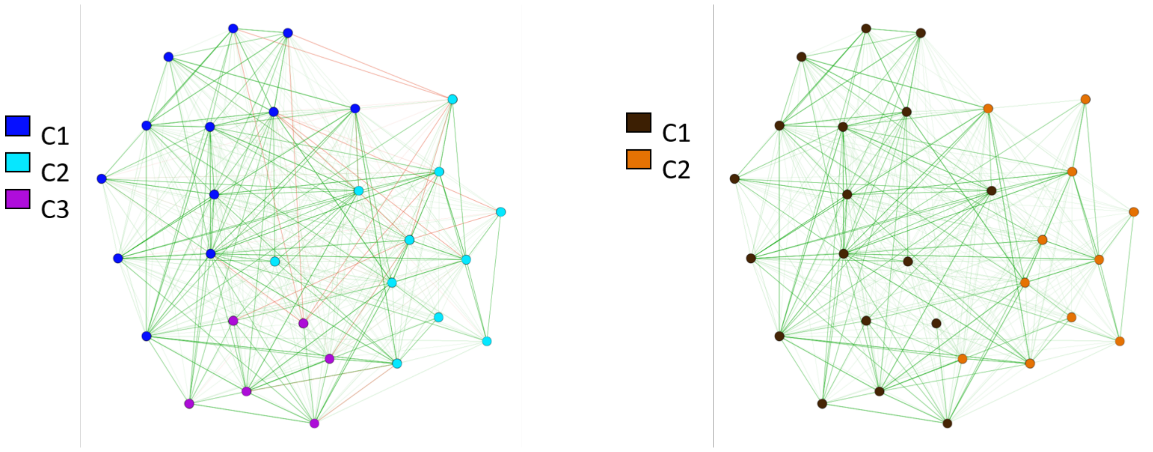

Figure S14: Community analysis of group A in wave 2 taking into account both positive and negative links (left) and only positive links (right). Nodes belonging to each community are marked by color as indicated in the plot. Friendship links are drawn in green, whereas enmity links are drawn in red.

	wave 1				wave 2			
**# positive links**	**0**	**1**	**2**	**3**	**0**	**1**	**2**	**3**
**option a**	0	15	106	2974	1	31	195	4774
**option b**	341	1403	1696	2974	1737	4974	2501	4774

Table S11: Number of observed triangles formed by a mix of positive and negative links in the first wave (left) and in the second wave (right) for the complete course depending on the criteria.

References

[1] Tamarit, I., Cuesta, J.A., Dunbar, R.I.M., Sánchez, A. Cognitive resource allocation determines the organization of personal networks. *Proc. Nat'l Acad. Sci. USA* **115**, 8316–8321 (2018).
